# Personalized Human Astrocyte‐Derived Region‐Specific Forebrain Organoids Recapitulate Endogenous Pathological Features of Focal Cortical Dysplasia

**DOI:** 10.1002/advs.202409774

**Published:** 2024-12-31

**Authors:** Jinhong Xu, Yufei Kong, Nawen Wang, Huijuan Li, Yunteng Li, Zhuo Liu, Yuling Yang, Xiao Yu, Huihui Liu, Jing Ding, Yi Wang, Rui Zhao, Zhicheng Shao

**Affiliations:** ^1^ Institute for Translational Brain Research State Key Laboratory of Medical Neurobiology MOE Frontiers Center for Brain Science Institute of Pediatrics National Children's Medical Center Children's Hospital Fudan University Shanghai 200032 China; ^2^ Department of Neurology Zhongshan Hospital Fudan University Shanghai 200032 China; ^3^ National Children's Medical Center Children's Hospital of Fudan University Shanghai 201102 China; ^4^ Shanghai Children' Hospital School of medicine Shanghai Jiao Tong University Shanghai 200062 China; ^5^ Department of Neurosurgery Children's Hospital of Fudan University Shanghai 201102 China

**Keywords:** disease modeling, focal cortical dysplasia, forebrain organoids, human astrocyte, induced pluripotent stem cells

## Abstract

Focal cortical dysplasia (FCD) is a highly heterogeneous neurodevelopmental malformation, the underlying mechanisms of which remain largely elusive. In this study, personalized dorsal and ventral forebrain organoids (DFOs/VFOs) are generated derived from brain astrocytes of patients with FCD type II (FCD II). The pathological features of dysmorphic neurons, balloon cells, and astrogliosis are successfully replicated in patient‐derived DFOs, but not in VFOs. It is noteworthy that cardiomyocyte‐like cells correlated with dysmorphic neurons are generated through the high activation of BMP and WNT signaling in some of the FCD‐organoids and patient cortical tissues. Moreover, functional assessments demonstrated the occurrence of epileptiform burst firing and propagative self‐assembling neuronal hyperactivity in both FCD‐DFOs and VFOs. Additionally, the heterotopic cardiomyocyte‐organoids demonstrated the capacity for cardiomyocyte contraction and rhythmic firing. The presence of these cardiomyocytes contributes to the hyperactivity of neural networks in cardioids‐DFOs assembly. In conclusion, the personalized region‐specific forebrain organoids derived from FCD patient astrocytes effectively recapitulate heterogeneous pathological features, offering a valuable platform for the development of precise therapeutic strategies.

## Graphical abstract

Region‐specific neural organoids derived from astrocytes of FCD II patients exhibited hallmarks of the disease, such as dysmorphic cells, epileptiform burst firing, and propagative self‐assembling neuronal hyperactivity. Notably, some organoids developed cardiomyocyte‐like cells, likely due to heightened BMP/WNT signaling. Furthermore, these heterotopic cardiomyocyte‐organoids showed the ability to contract and fire rhythmically. Cardiomyocyte‐like cells could also potentially lead to heightened neural activity.

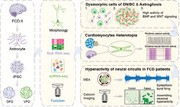

## 1 Introduction

Epilepsy is one of the most prevalent and heterogeneous neurological disorders, primarily distinguished by excessive paroxysmal discharges that are frequently spontaneous and recurrent. It is worth noting that up to 40% of medically refractory childhood epilepsy patients treated by surgery are attributable to the malformations of cortical development (MCDs).^[^ ^1^ ^]^ MCDs encompass a range of heterogeneous abnormalities affecting specific cortical structures. These include the main subtypes of FCD, hemimegalencephaly, and tuberous sclerosis complex (TSC).^[^ ^2^ ^]^ FCD is the most prevalent MCD subtype characterized by dyslamination, and the presence of dysmorphic neurons (FCD II a) and balloon cells (FCD II b).^[^ ^3^ ^]^ It has been postulated that balloon cells and dysmorphic neurons may respectively originate from radial glial cells and intermediate progenitor cells.^[^ ^4^ ^]^ FCD II is primarily associated with low‐level mosaic mutations in neural cells. It has been proposed that somatic cell mutations may occur in intermediate progenitor cells during the process of proliferation. These mutation‐carrying intermediate progenitor cells are then capable of giving rise to neurons that are similar to those carrying the mutations, which can ultimately result in the development of focal epilepsy.^[^ ^5^ ^]^ Moreover, caudal late interneuron progenitor (CLIP) cells have been demonstrated to undergo excessive proliferation, resulting in the formation of brain tumors and cortical malformations in TSC.^[^ ^6^ ^]^ Therefore, further investigation is required to elucidate the origin of the malformed neural cells, including dysmorphic neurons and balloon cells. The mTOR signaling cascade plays a pivotal role in regulating cell growth, proliferation, and metabolism in response to various environmental signals. During the process of brain development, excessive activation of the mTOR signaling cascade can disrupt the maturation and migration of neurons, leading to abnormal enlargement of neuronal cells and the formation of dysmorphic neurons, which are characterized by FCD II.^[^ ^7^ ^]^ It has been demonstrated that the dysregulation of the mTOR signaling pathway is associated with FCD II in ≈15.6% of patients.^[^ ^7^, ^8^ ^]^ Nevertheless, the molecular pathogenesis of the predominant cohort of FCD patients remains elusive. It is therefore imperative to develop a human FCD model to facilitate the exploration of precise therapeutic strategies.

Epilepsy has been conceptualized as a neural network anomaly that manifests pathologically as an excitatory‐inhibitory imbalance, resulting in neuronal hyperexcitability with excessive or highly synchronized discharges.^[^ ^9^ ^]^ An epileptic neural network comprises three key elements: the onset point, the propagation of epileptic activity, and the externally affected network.^[^ ^10^ ^]^ However, further investigation is necessary to gain a more precise understanding of the cellular mechanisms that underlie the initiation and propagation of the various abnormal and transient firing patterns observed in epileptiform neural networks.

Here, we reprogrammed human astrocytes isolated from epileptogenic zones of patients with refractory FCD II into induced pluripotent stem cells (iPSCs) and subsequently induced them to generate dorsal and ventral forebrain organoids including excitatory and inhibitory neurons for modeling the individual pathogenesis of patients. The FCD forebrain organoids exhibited a spectrum of endogenous pathological features, including dysmorphic neurons, balloon cells, aberrant astrogliosis, epileptiform burst firing, and self‐assembling neuronal hyperactivity. More interestingly, cardiomyocyte‐like cells were observed in some FCD‐organoids and patient cortical tissues, which correlated with dysmorphic neurons and were induced by highly activating BMP and WNT signaling. The heterotopic cardiomyocyte‐organoids displayed characteristics indicative of cardiomyocyte contraction and rhythmic firing. Furthermore, our findings confirm that cardiomyocytes contribute to the hyperactivity of neural networks in cardioids‐DFOs assemblies. The region‐specific forebrain organoid models developed in this study provide a valuable platform for the precise development of therapeutic strategies for patients with focal cortical dysplasia.

## 2 Results

### 2.1 Pathological Characterization of Focal Cortical Dysplasia and the Establishment of Personalized Patient‐derived iPSCs

To investigate the underlying mechanisms of developmental abnormalities in patients diagnosed with early‐onset refractory FCD II epilepsy, we isolated human primary astrocytes from their brain tissue samples and constructed iPSC lines (Figures S1A,B, Supporting Information). Magnetic resonance imaging (MRI) imaging confirmed the presence of focal epilepsy in all patients, with the identification of clear epileptic foci (Figure S1C, Supporting Information). The surgical resection of the brain tissue was subjected to hematoxylin‐eosin staining, which revealed the presence of typical dysmorphic neurons (DNs) and balloon cells (BCs) (Figure S1D, Supporting Information). Consequently, all patients, aged 2 months to 13 years, were diagnosed with FCD II refractory epilepsy and classified as having sporadic epilepsy with undefined genetic mutations, except patient 3, who had a somatic mutation in NPRL2 identified in blood cells and isolated astrocytes through whole‐exome sequencing (Figure S1B, Supporting Information). Our results presented that over 98% of the isolated cells expressed astrocyte markers GFAP and S100B (Figures S2A–D S2A‐D, Supporting Information). The electroporation of reprogramming factors was found to successfully reprogram astrocytes isolated from the focal brain regions of five patients and two control samples. One of the control samples was taken from a normal human fetal cortical brain, while the other was taken from a parafocal region. On day 23 after electroporation, it was observed that the reprogrammed cells had formed dense clones with distinct edges (Figure S2E, Supporting Information) and exhibited high expression of the pluripotent stem cell marker TRA‐1‐60 (Figure S2F, Supporting Information). Three to five iPSC lines were constructed and expanded for each patient and control (Figures S2F,G, Supporting Information). The expression of the pluripotency markers OCT4, SOX2, SSEA4, and TRA‐1‐60 in these iPSC lines indicated high pluripotency and all had normal karyotypes normal karyotypes (Figure S2H,I, Supporting Information).

### 2.2 Personalized Dorsal and Ventral Forebrain Organoids Mimic Endogenous Pathological Features of FCD II Patients

**Figure 1:**
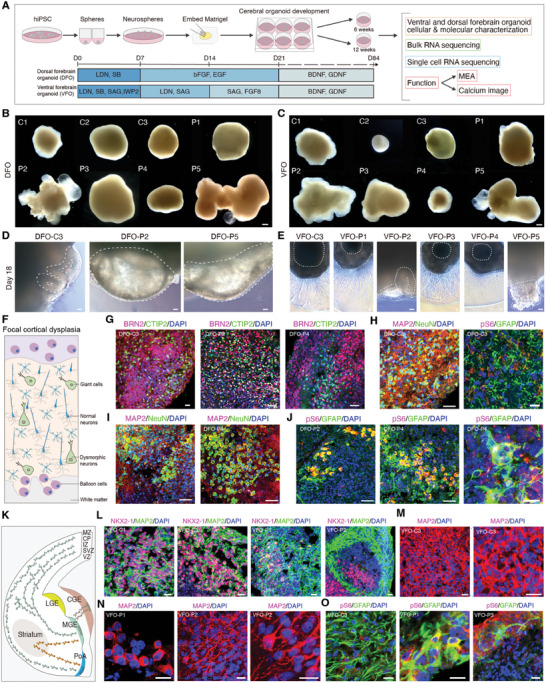
Personalized DFOs and VFOs display the pathological features of FCD II epilepsy. A) Summary pattern diagrams showing the individualized dorsal and ventral forebrain organoids derived from iPSCs of patients and controls, with their developmental status assessed using multi‐omics at 6 and 12 weeks. B, C) Representative images of DFOs (B) and VFOs (C) at 12 weeks reveal abnormal morphological features in the development of individual organoids. Scale bar, 500 µm. D) At day 18, forebrain organoids from controls exhibit normal neural tube chamber‐like structures, while P2 and P5‐derived DFOs show abnormal vesicular structures. Scale bar, 50 µm. E) The dendrites of inhibitory neuronal progenitor cells extend outward after embedding in Matrigel, while progenitor cells continue to proliferate abnormally in P2 and P5‐derived VFOs at day 18. Scale bar, 50 µm. F) Schematic representation of the pathological features of FCD epilepsy. G) Expression of the deep glutamatergic neuron CTIP2 and the superficial glutamatergic neuron BRN2 in 12‐week DFOs. Scale bar, 50 µm. H–J) Immunostaining shows the co‐expression of neuronal markers MAP2 and NeuN, balloon cell markers GFAP and pS6 in patient‐derived DFOs compared with controls, indicating abnormal dysmorphic neurons (H), balloon cells, and multinucleated cells (I, J). Scale bar, H and I: 50 µm; J: left and center: 50 µm, right: 20 µm. K) Displaying the origins of inhibitory neuronal migration and developmental patterns from MGE, LGE, and CGE. MGE, medial ganglionic eminence; CGE, caudal ganglionic eminence; LGE, lateral ganglionic eminence. L) VFOs express the inhibitory neuronal progenitor marker NKX2‐1 and the neuronal marker MAP2 at 6 weeks. Scale bar, 20 µm. M,N) Neuronal marker MAP2 immunostaining shows normal neuronal morphology in control‐derived VFOs both in patient and control. Scale bar, 20 µm. O) Immunostaining shows that astrocytes in patient‐derived VFOs co‐express GFAP and pS6, along with multinucleated cells. Right: the abnormal morphology is characterized by multinucleation. Scale bar, 20 µm.

To comprehensively investigate the pathogenesis of FCD, including the roles of excitatory and inhibitory neurons, we generated DFOs and VFOs and assessed their molecular, cellular, and neural functional characteristics at various stages comparing with normal control organoids from healthy iPSC line and normal human embryo stem cells (hESCs) H9 (**Figure** **1**A). Initially, we observed that the diameter of DFOs derived from some patients was significantly larger than that of the control DFOs, especially in those from patients 2 and 5, which exhibited abnormal cell proliferation (Figure 1B; Figure S3A, Supporting Information). Similarly, the diameter of VFOs at 12 weeks derived from some patients was significantly larger than that of the control VFOs (Figure 1C; Figure S3B, Supporting Information). To verify whether the enlargement of the organoids was due to the abnormal proliferation of neural progenitor cells, we sectioned and stained 6‐week‐old organoids. We only observed an increase in PAX6^+^ and SOX2^+^ neural progenitor cells in DFO‐Patient 2 (DFO‐P2) and DFO‐Patient 4 (DFO‐P4) compared to the controls (Figures S3C,D, Supporting Information). After 18 days of culture, control DFOs developed normally, forming typical neural tube structures. In contrast, DFO‐P2 and DFO‐P5 showed abnormal vesicular structures, suggesting abnormal cell proliferation (Figure 1D). We detected substantial axonal growth and cell migration in control VFOs as well as in VFO‐P1, ‐P3, and ‐P4. VFO‐P2 exhibited more neural tube‐like structures, but VFO‐P5 showed aberrant cell types with almost no neurites (Figure 1E). In summary, morphological structures of patient‐derived forebrain organoids demonstrated heterogeneous features.

We then examined whether patient‐derived DFOs exhibited cortical dyslamination, dysmorphic cells, and balloon cells (Figure 1F), which are the classical pathological features of FCD II patients. To explore the development of cortical layer neurons in DFOs and inhibitory interneurons in VFOs from both patients and healthy controls, we performed immunostaining on DFOs and VFOs at 6 and 12 weeks. We observed that patient DFOs expressed deep‐layer neuron markers TBR1 and CTIP2, as well as the superficial‐layer neuron marker BRN2, and all of them showed no differences compared to the controls (Figure 1G; Figure S3E, Supporting Information). However, we identified NeuN^+^ and MAP2^+^ dysmorphic neurons in 12‐week‐old DFO‐P2 and DFO‐P4, which were absent in the control DFOs (Figures 1H,I; Figure S3G, Supporting Information). The number and size of dysmorphic neurons were significantly increased in DFO‐P2 and DFO‐P4 (Figures S3F, Supporting Information). GFAP and pS6 co‐labelled balloon cells were found in DFO‐P2 and DFO‐P4, but not in control organoids, and DFO‐P4 particularly exhibited obvious multi‐nucleated astrocytes (Figures 1H,J). Furthermore, pS6 was highly expressed only in patient‐DFOs (Figures S3H, Supporting Information). Inhibitory interneurons originating from medial ganglionic eminence (MGE) are crucial for cortical development and excitation‐inhibition balanced neural network (Figure 1K). 6‐week‐old VFOs from both controls and patients showed expression of NKX2‐1, a specific MGE neural progenitor marker, as well as the neuronal marker MAP2 (Figure 1L; Figure S3I, Supporting Information). We did not find dysmorphic neurons in the 12‐week‐old VFOs. However, pS6/GFAP‐positive balloon cells and multi‐nucleated astrocytes were observed in VFO‐P1 and VFO‐P3, respectively (Figures 1M–O; Figure S3J, Supporting Information). These findings indicated that DFOs and VFOs derived from patient astrocytes could exhibit abnormal cell morphological features, including dysmorphic neurons and balloon cells.

### 2.3 Abnormal Gene Expression Profiles of Neuron and Cardiomyocyte in Patient‐Derived Organoids

**Figure 2:**
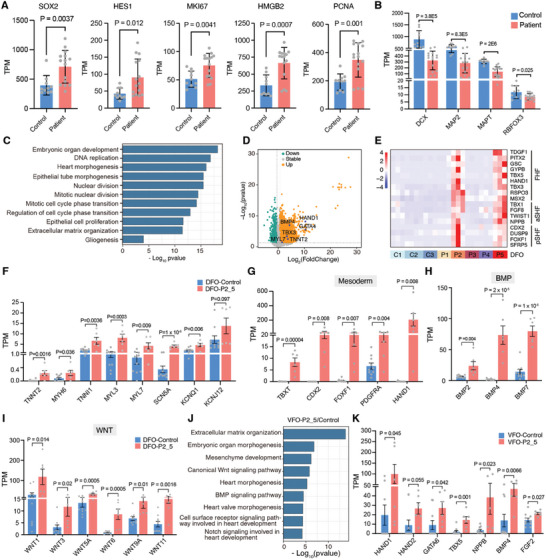
FCD patient‐forebrain organoids exhibit abnormal expression profiles of neuronal and cardiomyocyte genes. A,B) Graphs show the upregulation of neural progenitor markers and cycling progenitor markers, along with the downregulation of various neuronal marker genes in patient DFOs compared to controls (n = 3 independent experiments every patient and control). C) Graph showing GO term enrichment analysis of genes significantly upregulated (P < 0.05, log_2_FC > 0.58) in FCD II DFOs compared with controls. FC: fold change. D) Volcano plots visualize the differential gene expression results in patient DFOs and controls. X and Y axes show average log_2_FC and −log_10_ (P value). E) Cluster analysis of differentially expressed genes comparing patient DFOs with controls. Shown the first heart field (FHF) lineage, anterior second heart field (aSHF) lineage, and posterior second heart field (pSHF) lineage only significantly upregulated in patient 2 and 5‐derived DFOs. F–I) Graphs display the expression profiles of ion channels, structural proteins (F), mesoderm markers (G), BMP pathway markers (H), and WNT pathway markers (I) in control and patient 2‐ and 5‐derived DFOs (n = 3 independent experiments every patient and control). J) Graph showing GO term enrichment analysis of genes significantly upregulated in patient 2 and 5‐derived VFOs compared with controls. K) Expression of key genes for the development of cardiomyocytes in patients 2 and 5‐derived VFOs (n = 3 independent experiments every patient and control). A, B, F, G, H, I, K) Error bars are SEM of the mean; statistical significance was assessed using an unpaired two‐tailed *t*‐test.

To examine the transcriptional expression profiles of dorsal and ventral forebrain organoids induced from FCD II epilepsy, we conducted bulk‐RNA‐sequencing on DFOs and VFOs from patients and healthy controls and normal hESCs H9 at 6 weeks. Using principal component analysis (PCA), we observed distinct differences in the gene expression profiles between samples (Figure S4A, Supporting Information). Specifically, we identified 3954 differentially expressed genes (DEGs) in DFOs and 311 DEGs in VFOs (Figure S4B, Supporting Information). The gene expression profiles of DFOs were categorized into 13 modules through weighted correlation network analysis (WGCNA). The green module genes were primarily linked to cell cycle phase transition, whereas the yellow module genes, which are associated with cardiac chamber morphogenesis and development, were predominantly expressed in DFO‐P2 and DFO‐P5 (Figure S4C, Supporting Information). We found significant expression of neural stem cell markers SOX2 and HES1, cell proliferation markers MKI67, HMGB2, and PCNA, in patient‐derived organoids. However, the expression of neuronal markers such as MAP2, MAPT, RBFOX2, and DCX was significantly reduced in all of the patient DFOs (**Figures** **2**A,B). Gene ontology (GO) terms associated with the cell cycle, epithelial cell proliferation, and gliogenesis were enriched in patient DFOs (Figure 2C). Abnormal cytomegalic cells display a senescence‐like phenotype in FCD II surgical specimens.^[^ ^11^ ^]^ Previous studies have shown that MECP2 deficiency leads to over‐activation of the TP53 pathway and premature neuronal senescence.^[^ ^12^ ^]^ Here, we discovered that TP53 and TP73 genes, along with their target genes Puma (Bbc3) and Cdkn1a were upregulated in patient DFOs during early neuronal development (Figure S4E, Supporting Information). At the same time, MECP2 was significantly reduced, which may activate TP53‐mediated senescence pathways (Figure S4E, Supporting Information). Meanwhile, downregulated genes in patient DFOs, were involved in the synaptic transmission, synapse organization, and ion transport, indicating the neuronal defects (Figure S4F, Supporting Information). These results suggest that activation of the p53 pathway in patient DFOs may induce neuronal defects during the cortical developmental stage.

In addition to the neural developmental defects, we unexpectedly found that the upregulated genes enriched in patient DFOs were specifically involved in cardiomyocyte development (Figure 2C; Figure S4D, Supporting Information). The genes associated with cardiomyocyte development were significantly upregulated (Figure 2D). We observed that these genes specifically expressed in DFO‐P2 and DFO‐P5 were associated with the development of cardiac mesodermal progenitors in the first heart field (FHF) lineage (e.g., TDGF1, PITX2, GSC, HAND1) and the cardiac mesoderm lineage in the anterior second heart field (aSHF) (e.g., TBX1, TWIST1, NPPB) and posterior second heart field (pSHF) (e.g., CDX2, DUSP9, FOXF1, SFRP5) (Figure 2E). Additionally, genes encoding structural proteins expressed in mature cardiomyocytes (e.g., TNNT2, MYH6, TNNI1, MYL3, MYL7) and ion channels (e.g., SCN5A, CNQ1, KCNJ12) were significantly upregulated in DFO‐P2 and P5 (Figure 2F). Cardiomyocytes are derived from the mesoderm and are regulated by bone morphogenetic protein (BMP), fibroblast growth factor (FGF), and WNT signaling. We found that TBXT and PDGFRA, as mesodermal markers, were significantly activated in DFO‐P2 and DFO‐P5 (Figure 2G). Besides, BMP2, BMP4, and BMP7, and several WNTs (WNT1, WNT3A, WNT5A, WNT6, WNT9A, WNT11) were robustly upregulated (Figures 2H,I). To determine whether aberrantly developing cardiomyocytes were also present in patient VFOs, we performed GO enrichment analysis of the upregulated genes, which revealed significant enrichment in cardiac tissue development (Figures S4G,H, Supporting Information). In addition, markers associated with the development of embryonic progenitor cells in the heart (e.g., HAND1, HAND2, GATA6, TBX5, NPPB), and the signaling factors BMP4 and FGF2 were also significantly activated (Figures 2J,K). These findings suggest that some patient DFOs and patient VFOs with abnormal activation of BMP and WNT signaling pathways may aberrantly develop into mesodermal cardiomyocytes, leading to the formation of chimeric neuro‐cardiac organoids. In summary, our personalized dorsal and ventral forebrain organoid models could reveal the diversity of pathological features of FCD II patients.

### 2.4 FCD Patient‐Derived DFOs and VFOs Exhibit Ectopic Cardiomyocyte‐Like Cells and Abnormal Gliogenesis

**Figure 3:**
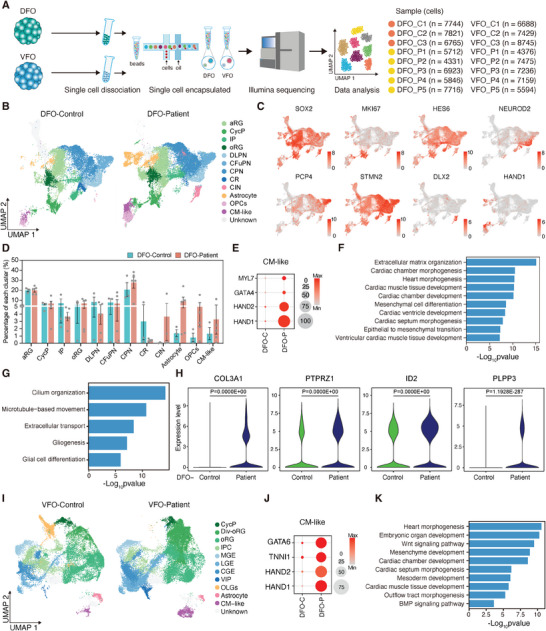
scRNA‐seq analysis reveals cardiomyocyte‐like cells and astroglial genesis. A) Workflow for single‐cell RNA‐seq of DFOs and VFOs derived from patients and controls. B) Cell clustering of DFOs derived from patients and controls integrated using Harmony and depicted using UMAP. Cells are colored by cell type: aRG, apical radial glial cell; CycP, Cycling Progenitor; IP, intermediate progenitor cell; oRG, outer radial glial cell; DLPN, deep layer progenitor neuron; CFuPN, corticofugal projection neuron; CPN, callosal projection neuron; CR, Cajal Retzius; OPCs, oligodendrocyte precursor cells; CIN, Cortical interneurons; CM‐like, Cardiomyocytes‐like cells. C) UMAP plots showing the gene expression of selected markers. The color scale shows normalized gene expression levels. D) Data separation by patient and control status revealed the proportion of each cluster in patient DFOs compared to controls. E) Dot plots showing the expression of key genes for the development of cardiomyocytes in CM‐like cells in patient DFOs compared to controls. The size of the circle reveals the percentage of cells expressing each gene per cluster. F) Graph showing Gene Ontology (GO) term enrichment analysis of genes significantly upregulated (P‐adjust < 0.05, |pct.1‐pct.2| > 0.1) in patient DFOs compared with controls. G) GO analysis of significantly upregulated genes in the astrocyte cluster of patient DFOs compared with controls. H) Genes associated with gliogenesis were significantly upregulated in patient DFOs compared with controls. The p‐value was determined using an unpaired two‐tailed *t*‐test. I) Cell clustering of VFOs derived from patients and controls integrated using Harmony and depicted using UMAP. Div‐oRG: Dividing oRG, OLGs: Oligodendrocyte. J) Dot plots showing the expression of key genes for the development of cardiomyocytes in CM‐like cells in patient VFOs compared to controls. The size of the circle reveals the percentage of cells expressing each gene per cluster. K) Graph showing Gene Ontology (GO) term enrichment analysis of genes significantly upregulated (P‐adjust < 0.05, |pct.1‐pct.2| > 0.1) in patient VFOs compared with controls.

To better identify the molecular and cellular variations in DFOs and VFOs of FCD patients, we performed single‐cell RNA sequencing. We analyzed 12‐week‐old DFOs and VFOs from patients and healthy controls, collecting 52858 cells from DFOs and 54702 cells from VFOs after quality control to exclude low‐quality cells (**Figure** **3**A). Data from DFO and VFO samples were integrated using the Harmony algorithm to minimize batch effects while preserving disease‐related differences (Figures S5A, S6A, Supporting Information). Using graph‐based Louvain clustering, we identified DEGs and annotated 12 distinct cell clusters in DFOs and 11 in VFOs based on these DEGs and related literature.^[^ ^13^ ^]^ We then performed dimensionality reduction using unified manifold approximation and projection (UMAP) (Figures S5B, S6B, Supporting Information). In DFO samples, cell clusters contained neuronal progenitors such as apical radial glial cells (aRGs), cycling progenitors (CPs), intermediate progenitors (IPs), and outer radial glial cells (oRGs). The clusters also contained excitatory neurons, including immature deep layer progenitor neurons (DLPNs), corticofugal projection neurons (CFuPNs), callosal projection neurons (CPNs), Cajal‐Retzius cells (CRs), and cortical interneurons (CINs), as well as astrocytes, oligodendrocyte precursor cells (OPCs) and cardiomyocyte‐like cells (CM‐like) (Figure 3B; Figure S5C, Supporting Information). We further analyzed the expression of genes that identify different cell populations in DFOs. Neuronal precursors were characterized by SOX2, PAX6, and MKI67. DLPNs were termed by NEUROD1 and NEUROD2; PCP4 and FOXP2 used for CFuPNs; STMN2 for CPNs; DLX2 for CINs; PDGFRA for OPCs; astrocytes via AGT and GJA1; and CM‐like cells by the marker HAND1 (Figure 3C; Figure S5C, Supporting Information). Particularly, we identified an abnormally developed cardiomyocyte‐like cell cluster that showed an increased percentage and exclusively expressed developmental cardiomyocyte‐related genes such as HAND1, HAND2, GATA4, and MYL7 in patient DFOs (Figures 3D,E; Figure S5D, Supporting Information). Through differential analysis of the CM‐like cell population, key regulatory genes involved in cardiomyocyte development were significantly upregulated. The differentially expressed genes were particularly enriched in pathways related to heart development and morphogenesis (Figure 3F; Figure S5E, Supporting Information). Taken together, these results confirmed that cardiomyocyte‐like cells may be ectopically generated in DFOs of FCD patients, which was consistent with the transcriptome sequencing data obtained at 6 weeks (Figures 2D–G).

We then performed differential gene expression analyses to compare the gene expression profiles between control and patient organoids. Patient DFOs showed significant enrichment of differentially expressed genes associated with epilepsy risk, representing 10.14% of upregulated genes and 12.82% of downregulated genes, which was confirmed using the DisGeNET epilepsy risk gene dataset C0014544 (Figures S5F,G, Supporting Information). Additionally, we observed that upregulated genes are involved in regulation of neurogenesis, such as NES, PTPRZ1, ZNF503, and TP53 related to the development and proliferation of neural progenitor cells were significantly expressed in patient DFOs (Figures S5H,J, Supporting Information). In excitatory neuron subclusters like DLPN, CFuPN, CPN, and CR, we found that genes linked to neurogenesis were upregulated, while those related to the glutamate catabolic process were downregulated (Figures S5I, Supporting Information). Interestingly, we noted an increased percentage of cortical interneurons co‐expressing BCL11B and DLX2 (Figure 3D; Figures S5K,L, Supporting Information). Furthermore, the formation of drug‐resistant epilepsy may be related to chronic neuroinflammation via the activation of glial cells.^[^ ^14^ ^]^ We found that the levels of astrocytes and oligodendrocyte progenitor cells were higher in patient DFOs compared to controls (Figure 3D). The significantly upregulated genes for gliogenesis were enriched in the astrocyte cluster in patient DFOs compared to the controls (Figures 3G,H). These results suggest that gliogenesis may exacerbate the occurrence and development of focal epilepsy.

In the VFOs, we sorted cells into 11 clusters based on the expression of differentially expressed and characterized marker genes (Figure 3I).^[^ ^15^ ^]^ These clusters included neural progenitors expressing TOP2A and HES1, intermediate progenitors expressing DLX2 and DLX5, region‐specific interneuron progenitor cells derived from the MGE expressing NKX2‐1 and SOX6, lateral ganglionic eminence (LGE) expressing MEIS2 and KLHL35, and caudal ganglionic eminence (CGE)S expressing NR2F1 and AP1S2. The clusters also included mature inhibitory neurons expressing VIP, oligodendrocytes expressing MBP, astrocytes expressing GJA1 and S100B, and cardiomyocytes expressing HAND1, TBX3, and GATA4 (Figures S6C,D, Supporting Information). Consistent with the 6‐week transcriptome sequencing results, we also detected cardiomyocyte‐like subsets in 12‐week VFOs. Notably, CM‐like cells were present in a higher percentage, and cardiomyocyte‐specific markers were exclusively expressed in patient VFOs (Figure 3J; Figures S6E–G, Supporting Information). Key genes and proteins for heart cell development, including HAND1, GATA4, TBX5, MYL7, TNNI1, KCNJ3, and BMP4, are highly expressed in organoids from patients but rarely expressed in control organoids (Figures S6H, Supporting Information). The upregulated genes in the CM‐like cluster were mainly associated with heart morphogenesis and the activation of the WNT and BMP signaling pathways (Figure 3K; Figure S6E, Supporting Information). It remains largely unclear whether inhibitory neuronal progenitors and neuronal subtypes are affected in the early development of FCD epilepsy. We observed an enrichment of DEGs associated with epilepsy risk in patient groups compared to controls. The upregulated genes (7.49%) and significantly downregulated genes (16.18%) were consistent with risk genes in the DisGeNET epilepsy dataset C0014544 (Figures S6I, Supporting Information). GO enrichment analysis comparing patients to controls in VFO or within inhibitory neuron subclusters showed that upregulated genes were primarily associated with chromatin remodeling and neurogenesis. In contrast, downregulated genes were associated with biological processes such as synapse organization and axon development (Figures S6J,K, Supporting Information). In summary, our results verified that both FCD patient DFOs and VFOs may show ectopic cardiomyocyte development via the activation of WNT and BMP signaling pathways, while gliogenesis was increased only in DFOs.

### 2.5 Assembling Neuronal Hyperactivity in FCD Patient DFOs and VFOs

**Figure 4:**
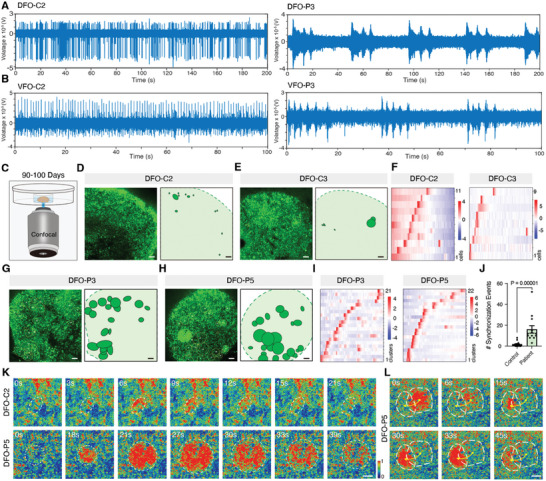
Epileptiform burst firing and assembling neuronal hyperactivity in FCD patients‐ DFOs and VFOs. A,B) Assessment of electrographic seizure‐like activities shows epileptiform burst firing in patient 3‐derived DFOs (A) and VFOs (B) compared to control 2 at 12–13 weeks using microelectrode array (MEA). C) Schematics of calcium imaging in organoids at 13–14 weeks using confocal microscopy. D–F) Calcium imaging shows firing activity in control DFO‐C2 and DFO‐C3 over 5 min (D, E), and a heat map displays single‐cell calcium transients across 100 recorded cycles (F). Scale bars, 100 µm. G–I) Calcium imaging shows firing activity in patient DFO‐P3 and DFO‐P5 over 5 min (G, H), with a heatmap displaying clustered cell calcium transients across 100 recorded cycles (I). Scale bars, 100 µm. J) Graph showing higher frequency of synchronization events in patient DFOs compared with controls (n = 3 independent experiments for every patient and control). The error bar is presented as mean ± SEM; The p‐value was determined using an unpaired two‐tailed *t*‐test. K) Fluorescence intensity graph showing the clustered cell firing process in patient DFO‐P5 over time, compared to the individual cell firing pattern in control DFO‐C2. Scale bars, 100 µm. L) Fluorescence intensity graph showing the characterization of the propagation of firing modes between two clustered cells in DFO‐P5. Scale bars, 100 µm.

To investigate the physiological activity of FCD brain organoids, we recorded neuronal network activity using microelectrode arrays (MEA). We initially recorded the extracellular electrophysiological activity of neurons in the DFOs and VFOs at 12–13 weeks. Analysis of the signals recorded from individual electrodes showed that the neurons in control organoids displayed typical electrical activity. However, spontaneous clusters of epileptiform burst firing with different patterns were observed in all the patient DFOs and some of the VFOs (**Figures** **4**A,B; Figures S7A,B, Supporting Information). Statistical analysis of electrophysiological activity showed that the number of spikes and active electrodes, and the number of network bursts were enhanced significantly in DFO‐P1, ‐P3, and ‐P4 compared to controls (Figure S7C, Supporting Information). In patient VFOs, we observed no significant difference in the number of inhibitory neuron spikes, and the mean firing rate decreased, except for the number of activated electrodes and network bursts in VFO‐P3 (Figure S7D, Supporting Information). These findings indicate that DFOs and VFOs derived from FCD patients exhibit abnormal epileptiform burst firing but with different firing intensities and patterns.

To further investigate the aberrant electrophysiological activity of neurons at the single‐cell level, we conducted calcium imaging to record spontaneous calcium activity in DFOs and VFOs at 13–14 weeks (Figure 4C). In the control groups, DFOs predominantly displayed spontaneous calcium activity in single neurons, with occasional synchronized clusters of calcium activity (Figures 4D–F). However, in FCD patient DFOs, we observed spontaneous neuronal clusters of synchronized calcium activity, which we termed as assembling neuronal hyperactivity (Figures 4G–I and Video S1, Supporting Information). The number of assembling neuronal hyperactivity events was significantly higher in FCD patient DFOs than in controls (Figure 4J). Interestingly, we found that the synchronized clusters of calcium activity started from a few neurons and gradually propagated to peripheral neurons, thereby increasing the range of synchronized activity (Figure 4K). Additionally, these clusters could trigger the transmission of calcium activity, leading to the next synchronized cluster, but this phenomenon is not present in control DFOs (Figure 4L and Video S1, Supporting Information). Furthermore, control VFOs mainly exhibited spontaneous calcium activity in single neurons (Figures S8A–C, Supporting Information). In contrast, FCD patient VFOs, which are predominantly composed of inhibitory neurons, also exhibited assembling neuronal hyperactivity (Figures S8D–F and Video S2, Supporting Information). The patient VFOs displayed significantly increased levels of synchronized assembling neuronal hyperactivity (Figure S8G, Supporting Information), which was also propagative (Figure S8H and Video S2, Supporting Information). Taken together, these findings demonstrated that the functional neuronal activity in the FCD patient DFOs and VFOs showed epileptiform burst firing and abnormal assembling neuronal hyperactivity of calcium activity with propagative features.

### 2.6 Functional Characteristics and Abnormal Developmental Mechanisms of Heterotopic Neuro‐Cardioid

**Figure 5:**
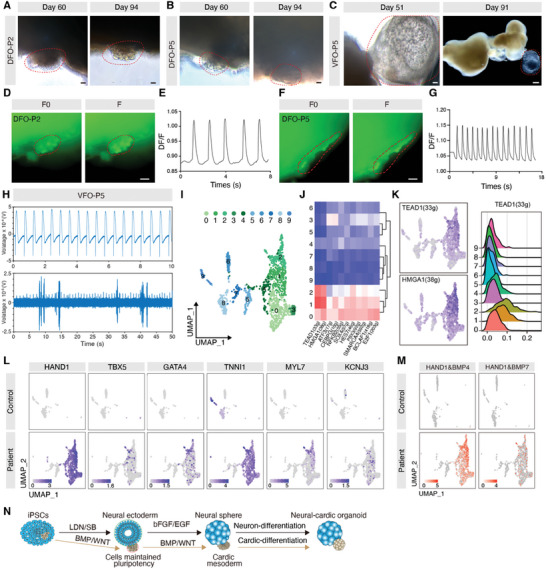
Functional characteristics and abnormal developmental mechanisms of heterotopic neuro‐cardioid. A,B) Showing the abnormally developed myocardial beating structures of DFO‐P2 (A) and DFO‐P5 (B) at days 60 and 94, with the region of myocardial beating structures circled by the red dashed box. Scale bar, 50 µm. C) Showing the abnormally developed myocardial beating structures of VFO‐P5 at days 51 and 91, with the region of myocardial beating structures circled by red dashed boxes. Scale bars, left: 50 µm, right: 500 µm. D) Fluo‐4‐AM fluorescence staining of DFO‐P2 neuro‐cardiac organoids was performed for calcium imaging and fluorescence intensity was analyzed as a function of time, left: background fluorescence, right: fluorescence of myocardial beating induced calcium transients. The Red dashed box represents myocardial beating structures. Scale bar, 50 µm. E) Statistics of fluorescence intensity changes of rhythmic calcium activity in cardiomyocytes in (D). F/F0: ratio of fluorescence change value to background fluorescence intensity. F) DFO‐P5 neuro‐cardiac organoids were fluorescently stained with Fluo‐4‐AM for calcium imaging, and fluorescence intensities were analyzed as a function of time, left: background fluorescence, right: fluorescence of beating‐induced calcium transients in cardiac myocardium. Scale bar, 50 µm. G) Counting of fluorescence intensity changes of rhythmic calcium activity in cardiomyocytes in (F). H) MEA recording of VFO‐P5 neuro‐cardiac organoids shows firing patterns recorded at single electrodes, with cardiomyocyte firing patterns at the top and neuronal firing patterns at the bottom. I) Cardiomyocyte subsets were separated from the DFOs, reclustered into 10 clusters, and visualized using UMAP. J) Single‐cell regulatory network analysis of the CM‐like cluster revealed some upregulated regulons in 10 clusters, g: genes. K) Left: UMAP plots represent TEAD1 (33g) and HMGA1 (38g) expression in the CM‐like cluster. Right: Ridgeline plot shows TEAD1 expression density in each CM‐like cell subcluster. g: genes. L) CM‐like cell subclusters from patient‐derived DFOs show abnormally developing cardiomyocyte genes that express cardiac development regulators HAND1, TBX3, and GATA4, as well as myocardial structural proteins TNNI3, MYL7, and KCNJ3. M) Co‐expression of HAND1/BMP4 and HAND1/BMP7 is present only in abnormally developing CM‐like clusters derived from patients. N) Diagram of abnormal developmental patterns of neuro‐cardiac organoids.

Both bulk and single‐cell transcriptome sequencing revealed abnormal developmental cardiomyocyte expression. To further investigate whether these specific cardiomyocyte‐like cells exhibit rhythmic beating and electrophysiological activities, we employed calcium imaging and MEA. We directly observed rhythmic beating in ≈16.6% of DFOs and VFOs from P2 and P5, starting as early as week 7 and persisting until week 13 (**Figures** **5**A–C and Video S3, Supporting Information). Additionally, we verified that these rhythmic beatings were accompanied by rhythmic calcium activity (Figures 5D–G; Figures S9A,B, Video S4, Supporting Information). Using the MEA module to record the electrical activity of cardiomyocytes, we observed a rhythmic firing pattern similar to that of the human heart. In the neuronal recording module, we detected concurrent electrophysiological activity from both neurons and cardiomyocytes, revealing a higher firing amplitude in neurons (Figure 5H). These findings suggest that the cardiomyocyte‐like cells in patient brain organoids exhibit typical characteristics of human cardiomyocytes with regular electrophysiological activity and spontaneous contraction.

To further investigate the lineage features and developmental mechanisms of aberrant cardiomyocytes in patient brain organoids, we isolated cardiomyocyte‐like clusters in DFOs and re‐clustered them into 10 subclusters (Figure 5I). Single‐cell regulatory network analysis of the CM‐like cluster revealed upregulation of certain regulons that were primarily activated in clusters derived from patient DFOs. Among these, TEAD1, a core component of the cardiac transcriptional network controlling cardiomyocyte‐specific gene functions,^[^ ^16^ ^]^ was particularly upregulated (Figures 5J,K). Genes related to the regulation of cardiac development, including HAND1, TBX5, and GATA4, were specifically expressed in the subset of cardiomyocytes derived from FCD patients. In addition, patient DFOs exclusively expressed the myocardial troponin protein TNNI1, the myocardial structural protein MYL7, and the ion channel protein KCNJ3 (Figure 5L). These findings demonstrated that abnormally developed cardiomyocytes in patient DFOs have similar gene expression profiles to normal cardiomyocytes. As previously mentioned, we found that the BMP signaling pathway which is essential for mesodermal and cardiomyocyte development was activated in patient DFOs at 6 weeks (Figure 2H). We also identified the co‐expression of BMP4 and BMP7 with HAND1, a germ layer marker in the heart (Figure 5M). Further transcriptome sequencing of focal brain tissue from patients P2 and P5 compared to P3 and P1 parafocal brain tissue (Ps1) also showed significant upregulation of BMP4 (Figure S9C, Supporting Information). Interestingly, we also found significant expression of pluripotency genes such as NANOG, POU5F1, and DPPA4 in DFO‐P2 and DFO‐P5 at 6 weeks (Figure S9D, Supporting Information). Additionally, single‐cell RNA sequencing of 12‐week‐old DFOs revealed the activation of pluripotency genes (Figure S9E, Supporting Information). Therefore, we proposed that during the induction of cortical organoids, some pluripotent cells were maintained due to the high activation of BMP and WNT signaling, and at a later stage, these cells aberrantly differentiated into mesodermal cardiomyocytes (Figure 5N).

**Figure 6:**
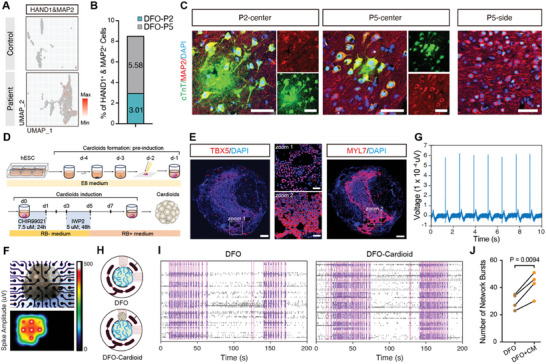
Validation of ectopic cardiomyocytes in pathological brain tissue and their impact on the neural network. A) Co‐expression of the cardiac mesodermal gene HAND1 and the neuronal marker MAP2 is observed only in abnormally developing CM‐like clusters derived from patients. B) Proportion of HAND1/MAP2 double‐positive cells in DFO‐P2 and DFO‐P5 (n = 3 organoids from one independent experiment every patient). C) Representative images of staining for the cardiac structural protein cTnT and the neuronal marker MAP2 in focal brain tissue samples from P2, P5, and in para‐focal brain tissue from P5. Scale bar, 50 µm. D) Diagram of cardioids induction. E) Cardiac mesoderm marker TBX5 (left) and myocardial structural protein MYL7 (right) staining in cardioids at 3 weeks. Scale bar, 200 µm (left and right), 50 µm (center). F) Displaying electrode recording images and spike amplitude heatmaps of cardioids using a microelectrode array. G) MEA recording of cardioids showed cardiomyocytes' rhythmically firing pattern at single electrodes. H) Schematic diagram of MEA recording comparing the spontaneous activities of single DFOs and DFOs integrated with cardioids. DFOs were derived from hESCs (H9 stem cell line). I,J) Raster plot showing spontaneous activity of DFOs and DFOs integrated with cardioids (I), with a higher number of network bursts observed in DFOs integrated with cardioids compared to single DFOs (n = 4 independent experiments), DFO+CM: represent DFO‐Cardioid (J). J) The p‐value was determined using a paired two‐tailed *t*‐test.

To further confirm these results, we observed co‐expression of HAND1 and MAP2 in 3.01% of DFO‐P2 cells and 5.58% of DFO‐P5 cells, suggesting the presence of chimeric cells with both neuronal and cardiac characteristics (**Figures** **6**A,B). Furthermore, focal brain tissue from patients P2 and P5 showed abnormal cardiac troponin cTnT expression in some MAP2^+^ neurons. These neurons generally had larger cell bodies compared to normal neurons, like dysmorphic neurons (Figure 6C). Additionally, the cardiac structural protein MYL7 was expressed in MAP2^+^ cells and co‐localized with troponin cTnT in focal brain tissue from P2 and P5, but not in P3 (Figures S9F,G, Supporting Information). Activation of the mTOR pathway in dysmorphic neurons resulted in the phosphorylation of its downstream factor pS6.^[^ ^1^, ^6^ ^]^ Our results also demonstrated that pS6 was co‐expressed with the cardiac troponin cTnT in the focal brain tissue of patients P2, P3, and P5 (Figure S9H, Supporting Information). To investigate whether abnormally developed cardiomyocytes are present in other patients with focal epilepsy, we collected astrocyte samples from three additional patients and induced them into brain organoids (Figure S10B, Supporting Information). Bulk RNA‐seq analysis also revealed aberrantly upregulated expression of cardiomyocyte development markers in the DFOs of two out of three patients (Figure S10A, Supporting Information). Additionally, we observed rhythmic calcium activity and contractions in DFO‐P6 at week 13 (Figure S10C and Video S5, Supporting Information). To further investigate whether the ectopic development of cardiac cells affects neural network activity, we generated human cardioids from hESCs (Figure 6D).^[^ ^17^ ^]^ These cardioids expressed the cardiac mesodermal progenitor marker TBX5 and the structural protein MYL7 and exhibited rhythmic contraction, discharge, and conduction, indicating their functionality (Figures 6E–G and Videos S6,S7, Supporting Information). We then combined dorsal forebrain organoids with human cardioids to mimic cardiomyocyte heterotopia and examined the neural network activity in these assembled organoids (Figure 6H). We observed an increase in neural network bursts in the DFO after integration with human cardioids (Figures 6I,J), suggesting that ectopic cardiomyocytes may increase neural activity. These results validate that ectopic cardiomyocytes may contribute to the dysmorphic cells in the brain of FCD patients and induce neural network dysfunction. In summary, our findings suggest that dysmorphic neurons of FCD patients may be chimeric neuro‐cardiac cells, which could aberrantly develop in the cortical brain via activation of BMP and WNT signaling and induce the dysfunction of the neural network, ultimately contributing to the pathogenesis of FCD epilepsy (Figure S11, Supporting Information).

## 3 Discussion

The pathogenesis of FCD epilepsy, including the origin of the dysmorphic neurons, balloon cells, and the formation of abnormal neuronal networks, remains elusive. In this study, we found that FCD patient astrocyte‐derived forebrain organoids recapitulate the dysmorphic neurons and balloon cells exhibit abnormal heterotopic cardiomyocytes and assemble neural hyperactivity through prominent activation of BMP and WNT signaling pathways. Although patient iPSC‐derived 3D models have been utilized in various studies to investigate epilepsy mechanisms, these studies have predominantly focused on diseases characterized by well‐defined genetic mutations and clinical features, such as Rett syndrome,^[^ ^18^ ^]^ Angelman syndrome,^[^ ^19^ ^]^ Timothy syndrome,^[^ ^13a^ ^]^ TSC,^[^ ^6^, ^20^ ^]^ and Miller‐Dieker syndrome.^[^ ^21^ ^]^ Brain organoid models, as mentioned above, have been employed to reveal the mechanism of epilepsy, such as ion channels, abnormal migration of interneurons, amplification of CLIP cells, and prolonged proliferation of oRG. However, 30–70% of MCD cases are highly heterogeneous and still involved in unknown genetic mutations.^[^ ^8^, ^22^ ^]^ Without an efficient personalized human model, there has been restricted research on these particular types of disease. Therefore, we primarily focus on sporadic FCD epilepsy and utilize endogenous astrocyte‐derived region‐specific forebrain organoids to mimic the individualized pathogenesis of patients. It has been reported that the unstable cohesion of neuroepithelial cells influences the developmental delay of progenitor or newborn neurons, which hinders the formation of neural networks in FCD iPSC‐derived cortical organoids.^[^ ^23^ ^]^ In our study, we first reported the cardiomyocyte heterotopia in the epileptic model of DFOs and VFOs in FCD patients. Cardiomyocytes develop from the mesoderm regulated by BMP and WNT signaling.^[^ ^24^ ^]^ Brain tissue from FCD patients showed high levels of BMP4 in malformed neurons, such as dysmorphic neurons, giant neurons, balloon cells, giant cells, and reactive astrocytes.^[^ ^25^ ^]^ In our data, we also found that high levels of BMP and WNT signaling were maintained in dorsal forebrain organoids and patient cortical tissue. Furthermore, some cells within the patient organoids retained their pluripotency during neural differentiation. Thus, our results propose that this abnormal development of cardiomyocytes results in the differentiation of residual pluripotent cells into cardiomyocytes due to maintaining high levels of BMP and WNT signaling. This FCD organoid model may provide a pivotal platform for screening new drugs via targeting the inhibition of the BMP/WNT signaling pathway without toxicity to healthy neural cells.

Previous reports showed that ClueGO analysis of MCD risk genes was found to enrich cardiac muscle cell contraction.^[^ ^22a^ ^]^ In the spatial transcriptomic analysis of the human embryonic cerebral cortex at 23 weeks, the expression of several cardiac‐related genes, including MYL7, TNNT2, and ACTC1, was observed in the superior temporal lobe (ST). Additionally, co‐localization between TNNT2 and neural marker NEUROD2 was shown in ST.^[^ ^26^ ^]^ In a case report study, heterotopic myocardial tissue in the right superior temporal gyrus was reported in a 73‐year‐old woman with hemiplegia. It has been suggested that heart muscle tissue heterotopically develops into the brain during the embryonic period in the form of an abnormal interstitial component.^[^ ^27^ ^]^ Cardiomyocytes can supply nerve growth factors to sympathetic nerves via the neuro‐cardiac junction, which is essential for heart sympathetic innervation.^[^ ^28^ ^]^ All these studies, combined with our results, may indicate that cardiomyocytes might ectopically develop in the cortical brain of FCD patients during the early stages of brain development via aberrant activity of morphogens. Heterotopic CM‐like cells may interact with neurons and ultimately contribute to the cellular pathogenesis of FCD epilepsy. In addition, it remains to be shown, whether the presence of cardiomyocytes in the brain affects FCD epilepsy. There are still several questions that need in‐depth investigation, for instance how cardiomyocytes may further influence the development or functional changes in the brain. In the future, it may be necessary to transplant these abnormally developed cardiomyocytes into the mouse brain to verify their effect on the function of the cortical neural networks.

Furthermore, these organoids derived from patient astrocytes can recapitulate the characteristics of epileptiform discharges. Importantly, we observe the assembling neuronal hyperactivity of calcium activity in both the dorsal and ventral forebrain organoids using spontaneous calcium recordings. This single assembling neuronal hyperactivity causes the propagation of the epileptic discharge. It has been reported that in vitro‐induced 3D cortical brain organoids detect complex oscillatory waves through MEA recording, similar to the electroencephalogram characteristics of premature infants.^[^ ^29^ ^]^ Individualized brain organoids in Rett syndrome simulate highly abnormal epileptiform activity. By using an unconventional neuromodulating drug, Pifithrin‐alpha, the key physiological activity was successfully restored.^[^ ^18^ ^]^ A seizure occurs when there is a significant change in brain activity patterns, typically characterized by excessive and synchronized neuronal activity within a large population of neurons.^[^ ^30^ ^]^ Using MEA recording, we have also recorded epileptic discharges that resemble epileptic discharges in the brain.^[^ ^31^ ^]^ In the epileptic mouse model, epileptic networks display more network events that are composed of multiple synchronous cell clusters.^[^ ^32^ ^]^ Here, we have shown for the first time that multiple assembling neuronal hyperactivities of calcium activity in FCD organoids are consistent with those found in mouse models. Seizures often originate in localized epileptic foci and can then spread secondarily throughout the brain.^[^ ^33^ ^]^ Neuronal assemblies are initiated and become synchronous cell clusters, which then spread to neighboring clusters. The assembling neuronal hyperactivity of calcium activity is more similar to the abnormal neural network activity of epilepsy patients, which could be a powerful indicator for drug screening in individualized organoid models in the future. The grafted human brain organoids could mature and integrate into the neural networks of host brains in animal models, providing a powerful tool for studying human brain development and diseases.^[^ ^34^ ^]^ In future studies, transplanting each FCD patient‐derived organoid into the mouse cerebral cortex may further investigate in‐vivo cell developing trajectory and confirm pathological features of FCD such as heterotopic CM‐like cells and increased neuronal hyperactivity which may induce abnormal behaviors in mice.

Collectively, we found multiple and heterogeneous pathological features including dysmorphic neurons, balloon cells, astrogliosis, cardiomyocyte‐like cells, and assembling neuronal hyperactivity in our FCD patient region‐specific organoid models via maintaining high levels of BMP and WNT signaling. Our findings may suggest that cardiomyocytes may generate heterotopically in the cortical brain and transform into malformed neurons causing the hyperactivity of neural circuits in FCD patients (Figure S11, Supporting Information). Cardiomyocyte heterotopia and the propagation of assembling neuronal hyperactivity may serve as the precise therapeutic targets for FCD patients in the future.

## 4 Experimental Section

### Disassociation of Mature Human Astrocytes

Surgically excised human brain specimens were obtained from patients diagnosed with FCD II. The focal brain tissues were dissected in 6 cm petri dishes containing DPBS. The grey matter was then carefully removed and the meninges and blood clots were removed. A scalpel blade was then utilized to chop the brains into pieces smaller than 1 mm^3^. Approximately 0.5 g of tissue was placed into each 6 cm petri dish, and an Enzyme stock solution supplemented with 20 U mL^−1^ papain (Worthington, LS 03126), L‐cysteine hydrochloride monochloride (Sigma, C7880), and 10 ug mL^−1^ DNase I was added. The tissue was incubated for 100 min at 37 °C in an oxygenated atmosphere. After digestion, the digested tissues were transferred into a universal tube. After allowing the tissues to settle, the supernatant was carefully aspirated. Inhibitor stock solution was then added to the cells for washing, and once again, the tissue was allowed to settle again. This washing procedure was repeated a total of four times. Add 5 mL of astrocyte medium (AM) (Catalog 1801, Sciencell) to the universal tube. Using a serological pipette, aspirate the brain tissue together with the AM and release it rapidly, repeating this process 30–40 times. The AM will become cloudy, allowing pieces of tissue to settle. Using a 1 mL pipette, carefully transfer the single cells located on top of the chunks to a Falcon tube. Add 5 mL of AM to the universal tube and repeat the trituration process. It is worth noting that grey matter will dissociate at a faster rate compared to white matter. Cease trituration when only white matter remains visible among the brain pieces. The digested single‐cell suspension was subjected to two rounds of filtration using a 30‐micron cell strainer. After filtration, the suspension was centrifuged at 300 g for 5 min. The single‐cell solution was cultured in plates coated with Matrigel (Catalog 365230, BD), using an AM. The protocol for collecting astrocytes was approved by the Scientific Research Sub‐Committee of the Medical Ethics Committee at Children's Hospital, affiliated with Fudan University. The ethical protocol approval number is NO.(2021)78.

### Whole‐Exome Sequencing

Genomic DNA was extracted from blood leukocytes of patients 1–5, astrocytes derived from surgically resected brain tissue of patients 1–5, and parafocal astrocytes isolated from parafocal tissue of patient 5. First, the germination and Mosaic variation of mTOR pathway‐associated genes were detected by sequencing the whole exome. Collect and concentrate using SureSelect V6‐post according to the manufacturer's instructions. The samples were sequenced using HiSeq 2500 and NovaSeq Illumina platforms with 150 bp reads to achieve 100x coverage (≈10 Gb/sample) for blood leukocytes from patients 1–5, and 300x coverage (≈30 Gb/sample) for astrocytes derived from surgically resected brain tissue of patients 1–5, as well as parafocal astrocytes isolated from parafocal tissue of patient 5. In the genome analysis toolkit, with the help of BAM alignment, processing, and detection of workflow variants based on GRCh37's reference to the human genome, germline and mosaic variants were selected with priority given to stopping codon, frameshift, missense, nonsense and splicing site mutations, variants whose minor allele frequency is < 0.01, variants absent in gnomAD (Exome Aggregation Consortium) and in a publicly available genomic database of Brazilian samples. When < 10% of the readings were not aligned to the human genome reference, the variation is classified as a Mosaic mutation.

### Generation of Personalized iPSCs

Human astrocytes derived from individuals with FCD II were cultured and expanded in an AM. To generate iPSCs, a 1:1:1 ratio of the following episomal plasmids was used for transfection: pCXLE‐hOCT3/4‐shp53‐F (Addgene, 27077), pCXLEhSK (Addgene, 27078), pCXLE‐hUL (Addgene, 27080), and pCXLE‐EGFP (Addgene, 27082). Electroporation was performed with 4.5 µg of the plasmid mixture, using Nucleofector 2b system (Amaxa, Lonza), and following the instructions in the Nucleofector kit manual (NHDF, Lonza). The T‐020 system was employed for selection purposes. Following transfection, the cells were seeded onto Matrigel‐coated 35 mm plates and cultured in AM for 2 days. Subsequently, the medium was switched to Essential 8 (E8, Gibco) medium until colonies of human iPSCs formed. For each patient‐derived astrocyte, five monoclonal cell lines were selected.

### Maintenance of Personalized iPSCs

Under feeder‐free conditions, personalized iPSCs were cultured. These feeder‐free iPSCs were seeded onto 6‐well plates that were pre‐coated with Matrigel, and they were maintained in E8 medium. The cells were fed daily and passaged every 3–4 days using TrypLE solution for cell detachment, followed by neutralization with DMEM containing 10% FBS (Fetal Bovine Serum).

### Karyotype Analysis

Karyotype analysis was performed on five iPSC cell lines derived from patients, one from a human fetal cortical astrocyte, and one from a patient 5 parafocal astrocyte. The iPSC cell lines requiring karyotype analysis were inoculated on 6 cm Petri dishes and cultured until they reached 80–90% confluence. The cells were treated with colchicine for 4 h before harvesting, followed by hypoosmotic treatment in a 37 °C water bath for 15 min after digestion of the clones, and then fixed for subsequent imaging.

### Generation of Personalized Dorsal and Ventral Forebrain Organoids

Personalized iPSCs were cultured in an E8 medium on Matrigel‐coated plates. Subsequently, the cells were detached using TrypLE (Gibco) and transferred to a low‐attachment 96‐well plate (Corning) to grow as spheroids. The spheres were seeded at a density of 10000 cells per sphere.

During the dorsal forebrain organoid induction protocol, the neural induction phase from day 0 to day 7 entailed culturing the spheres in KSR medium (consisting of DMEM, 20% knockout serum replacement, 2 mm L‐glutamine, and 10 µm β‐mercaptoethanol). The medium was further supplemented with LDN193189 (100 nm), SB431542 (10 µm), and a ROCK inhibitor Y27632 (10 µm) on the first day. On the following day, the KSR medium was supplemented with LDN193189 (100 nM) and SB431542 (10 µm) and continued to be cultured until day 7. During the neural progenitor cell (NPC) proliferation phase from day 8 to day 21, the culture medium was switched to B27 medium (comprising Neurobasal, 2% B27, 100X NEAA, and 100X GlutaMax). The medium was further supplemented with 20 ng mL^−1^ bFGF and 20 ng mL^−1^ EGF. For neural maturation starting from day 22, the medium was changed to B27 medium supplemented with 10 ng mL^−1^ GDNF, 10 ng mL^−1^ BDNF, and 200 µm ascorbic acid.

During the ventral forebrain organoid induction protocol, the spheroids were cultured in KSR medium supplemented with LDN193189 (100 nM), SAG (0.1 µm), SB431542 (10 µm), and IWP2 (5 µm) from day 0 to day 7. On the first day, a ROCK inhibitor, Y27632 (10 µm), was also added to the medium. From day 8 to day 14, SB431542 and IWP2 were excluded from the medium. Subsequently, from day 15 to day 21, the organoids were cultured in a B27 medium supplemented with SAG (0.1 µm) and 100 ng mL^−1^ FGF8. Starting from day 22, the medium was changed to B27 medium supplemented with 200 µm ascorbic acid, 10 ng mL^−1^ GDNF, and 10 ng mL^−1^ BDNF to promote the differentiation of NPCs into interneurons. Throughout the protocol, the medium was refreshed every 2 days.

### Cardioid Induction

On day 4, seeding 5000 hESC H9 in Ultra‐Low Attachment 96‐Well Plates, each well‐containing E8 medium with 10 µm Y‐27632. After centrifugation of 500 g for 3 min, the plates were incubated at 37 °C to promote cell aggregation into spheres. After 24 h, the medium was replaced with E8 medium. The resulting spheres were embedded with 20 µL of Matrigel and incubated at 37 °C for 30 min to allow for coagulation on day 2. Subsequently, the embedded spheres were transferred to the E8 medium, and a medium change was performed after an additional 24 h. On day 0, The cardioid was initially induced with CHIR99021 (7.5 µm) in RB‐ (RPMI/1640 medium containing 2% B27 without insulin) and replacing RB‐ after 24 h. On day 3, IWP2 (5 µm) was added to RB‐ to induce cardioid formation for 48 h and then switched to RB‐ on day 5. The cardioid was initially transferred to a 24‐well plate in a shaker and cultured in RB+ (RPMI/1640 medium containing 2% B27) from day 7.

### Calcium Imaging in Intact DFO and VFO

After incubating intact DFO and VFO from day 90 to 100, a 10 µm solution of Fluo‐4 acetoxymethyl ester (Fluo‐4 AM, Invitrogen) was added and left for 30 min in neural medium. Subsequently, the samples were washed with a neural medium for 15 min. Calcium transient imaging was performed using an Olympus confocal microscope equipped with a resonant scanner. Spontaneous calcium activity was recorded for 100 cycles at a resolution of 512 pixels, capturing one frame every 3 seconds. The fluorescence intensity (F) was exported as mean grey values in ImageJ, and to control signal decay, the mean fluorescence of the background (Fb) was subtracted. To determine fluctuations in intracellular calcium, ΔF was calculated using the formula (Fcell – Fb)/F0, where F0 represents the minimum F value per cell recorded during the entire 100 cycles, after subtracting Fb. When a minimum of three neurons exhibit simultaneous calcium activity, refer to it as synchronized activity.

### Multielectrode Array Recording for DFOs and VFOs

To detect neuronal network activity, MEA detecting systems were employed. Each well of the 6‐well MEA plates (Axion Biosystems, Atlanta, GA, USA) was equipped with 64 low‐impedance platinum microelectrodes (0.04 MΩ/microelectrode) having a diameter of 30 µm and spaced 200 µm apart. The plate was precoated with a 1:100 dilution of Matrigel and incubated for 30 min. The mature DFO and VFO organoid was placed in a coated MEA well. Matrigel was added to immobilize organoids and incubated for 10 min, followed by the addition of fresh Neural Medium. Recordings lasting 10 min were conducted using a Maestro pro‐MEA system and analyzed using the AxIS Software Spontaneous Neural Configuration (Axion Biosystems). For data analysis, the Neural Metric Tool (Axion Biosystems) was utilized to classify active electrodes, which were defined as electrodes detecting at least 5 spikes per minute. Spike bursts were identified based on an inter‐spike interval (ISI) threshold, requiring a minimum of 5 spikes with a maximum ISI of 100 ms.

### Fixation and Cryo‐Sectioning of Organoids and Focal Brain Tissues

Dorsal and ventral forebrain organoids focal brain tissues were fixed overnight at 4 °C in a 4% paraformaldehyde (PFA) solution. Following fixation, the organoids and tissues were rinsed three times with PBS and subsequently immersed in PBS containing 30% sucrose to facilitate sinking. The next step involved embedding the organoids and tissues in the OCT compound (Tissue Tek, 4583) and placing them in a container. The organoids and tissues were then snap‐frozen and stored at −80 °C. To prepare the organoids and tissues for analysis, they were sequentially sectioned at a thickness of 20–30 µm using a Leica cryostat (model CM3050S). These sections were carefully mounted onto glass slides intended for microscopy, left to dry overnight, and stored at −80 °C. These prepared sections were now ready for subsequent immunohistochemical analysis.

### Immunostaining and Imaging

Immunocytochemistry was performed to evaluate the pluripotency of personalized iPSC cells. The cells were cultured on coverslips with E8 medium until dense colonies formed, followed by fixation with a 4% PFA solution for 15 min at room temperature. After fixation, coverslips underwent three 10 min washes with PBS. Subsequently, they were blocked for 20 min using a solution of PBS containing 3% BSA and 0.3% Triton X‐100. Following the blocking step, the cells were washed three times with PBS for 10 min each. For primary antibody staining, the cells were incubated overnight at 4 °C with antibodies against pluripotency markers, including OCT4 (1:5000), SOX2 (1:500), SSEA4 (1:100), and TRA‐1‐60 (1:100), diluted in PBS containing 3% BSA and 0.3% Triton X‐100. After incubation, the cells were washed three times with PBS for 10 min each. Next, the cells were incubated with secondary antibodies in PBS containing 3% BSA for 1 h at room temperature. To visualize the cell nuclei, DAPI staining was performed.

To prepare organoid and focal brain tissue sections for analysis, they were permeabilized and blocked in a solution of PBS containing 3% BSA and 0.3% Triton X‐100 for 1 h at room temperature. Before this step, the sections underwent extensive washing with PBS. Following the permeabilization and blocking process, the sections were incubated with primary antibodies that were appropriately diluted in an antibody solution consisting of 3% BSA and 0.3% Triton X‐100. The incubation was carried out overnight at 4 °C. Following three 10‐min washes with PBS, the sections were incubated with secondary antibodies, diluted in an antibody solution, at room temperature for 1 h. Subsequently, the sections were treated with a DAPI solution (2 mg mL^−1^) for 10 min.

Afterward, both coverslips, organoid, and focal brain tissue sections underwent three washes with PBS and were subsequently mounted in a fluorescent mounting medium. The primary antibodies utilized in this study, along with their respective dilutions, are outlined in Table S1 (Supporting Information). For secondary antibodies, AlexaFluor 488, 568, or 647‐conjugated donkey antibodies (Invitrogen) were employed at a dilution of 1:500. Immunostaining images were captured using Olympus confocal microscopes.

### Bulk RNA‐Sequencing

To compare the dorsal and ventral forebrain organoids with the fetal cortex and patient parafocal tissue astrocyte‐derived control organoids, three replicates were employed for each personalized organoid group. This comparison was conducted on day 42 of the experiment.

The RNA extraction of organoids was performed using RNAiso Plus (Takara, 9109), following the manufacturer's instructions. The concentration and quality of RNA were assessed using a NanoDrop 2000 spectrophotometer (Thermo Scientific, USA). Furthermore, the RNA integrity was evaluated using the Agilent 2100 Bioanalyzer (Agilent Technologies, Santa Clara, CA, USA). Subsequently, the libraries were prepared using the TruSeq Stranded mRNA LT Sample Prep Kit (Illumina, San Diego, CA, USA), following the provided manufacturer's guidelines. The libraries were subjected to sequencing using a HighSeq 2500 instrument (Illumina), which generated paired‐end reads of 150 bp. The mRNA sample isolation, library preparation, and sequencing processes were conducted at OE Biotech Co., Ltd (Shanghai, China). The raw data in fastq format underwent preprocessing using Trimmomatic, resulting in ≈40–65 million raw reads per sample. These raw reads were filtered to obtain clean reads by removing sequences containing adapters, poly‐N sequences, and low‐quality reads. The resulting clean reads were subsequently utilized for downstream analyses. Aligning the clean reads to the Homo sapiens genome (hg38) was performed using HISAT2, and HTSeq‐count was employed to count the aligned reads.

For the differential expression analysis, DESeq2 (v1.16.1) was utilized to analyze the samples. Additionally, the reads underwent TPM (transcripts per million) estimation using Kallisto (v0.43.0). The DESeq2 datasets were filtered based on a log2 fold change absolute value of ≥ 1 and an adjusted p‐value < 0.05. To explore gene expression patterns, hierarchical cluster analysis was performed on the DEGs. Furthermore, R‐based GO enrichment and KEGG pathway enrichment analysis were separately conducted on the DEGs, employing the hypergeometric distribution. Lastly, highly connected clusters were identified using the ClusterONE plug‐in within the Cytoscape software.

### Single‐Cell RNA‐Sequencing

To investigate lineage changes during cell development in patient‐derived DFO and VFO at day 84, single‐cell RNA‐seq was performed using the 10x Genomics platform. For population analysis, 2–3 organoids were transferred to a 35 mm dish containing fresh Neural Medium. The organoids were then fragmented into small pieces. These organoid pieces were further dissociated into single cells by incubating them in a Papain‐based solution containing Papain (20 Unit mL^−1^, LS03126, Worthington), DNase I (10 µg mL^−1^, 11284932001, Sigma), and L‐cysteine hydrochloride monochloride (180 µg mL^−1^, C7880, Sigma) at 37 °C with gentle shaking (300 rpm) for 45 min. To obtain single‐cell suspensions, the mixture was regularly pipetted using a glass Pasteur pipette. Following the dissociation, the cells were centrifuged at 300 g for 5 min and resuspended in ice‐cold PBS. Subsequently, they were passed through a 40 µm filter and kept on ice. The final cell density was adjusted to 300–600 cells µL^−1^.

To form Gel Bead‐In‐Emulsions (GEMs), a mixture containing dissociated single cells, enzymes, and barcode‐labeled beads was enclosed within oil droplets and loaded onto a Chromium Single Cell 3′ chip. Within the GEMs, the cells underwent lysis and reverse transcription, resulting in the generation of cDNA products. These cDNA products were then tagged with barcodes for identification. Subsequently, the GEMs were broken, enabling PCR amplification of the cDNA templates. The quality of the amplification products was assessed using Agilent 4200. To prepare single‐cell RNA‐seq libraries, the 10x Genomics Chromium Single Cell 3′ Library and Gel Bead Kit v3 were employed. Sequencing was performed on the Illumina NovaSeq6000 platform, generating paired‐end 150 bp reads. These reads were aligned to the human reference genome (GRCh38) using Cell Ranger. Barcode filtering and Unique Molecular Identifier (UMI) counting were carried out to process the data. Further analysis of the reads, including tallying for each feature in each cell, was conducted using the R package Seurat. The single‐cell RNA sample isolation, library preparation, and sequencing processes were conducted at OE Biotech Co., Ltd (Shanghai, China).

### Preprocessing of scRNA‐Seq Data

To ensure data quality and consistency, genes expressed in fewer than ten cells were filtered out. Additionally, cells with less than 1000 detectable genes, fewer than 500 reads, or a mitochondrial rate higher than 10% were excluded. Following this, the gene expression was normalized across the cells using the “LogNormalize” global‐scaling normalization method. The data was then integrated and scaled by a factor of 10000. Proceeded with downstream dimensionality reduction techniques, creating a UMAP plot for visualizing cell clustering. This plot was overlaid, and a differential expression analysis was conducted using the Seurat R package. For PCA, the top 3000 highly variable genes were selected, which were then scaled. In an unsupervised approach, single‐cell samples were clustered based on their gene expression profiles. To minimize technical variation across different batches, the R package Harmony was utilized and selected the top 30 principal components. A shared nearest neighbor graph was constructed using the k‐nearest neighbors (KNN) algorithm, calculated from the first 30 principal components of the scaled data with k set to 20. This step was executed using the FindNeighbors function in Seurat. The number of clusters was determined using a modularity function optimizer based on the Louvain algorithm, with a resolution parameter set to 0.8. This was accomplished using the FindClusters function within Seurat. Additionally, data reduction was carried out using UMAP. In the final step, cell clusters of the dorsal and ventral forebrain organoid were classified based on the expression patterns of established markers specific to the dorsal and ventral forebrain cortex.

### Identification of Differentially Expressed Genes

To compute the DEGs between Patient‐DFO/‐VFO and Control‐DFO/‐VFO cells, the Wilcoxon Rank Sum test via the “FindAllMarkers” function was employed within the Seurat R package. This analysis was conducted with parameters set to thresh.use = 0.25 and test.use = “bimod”, enabling to pinpoint variations in gene expression across the two cellular groups. DEGs were delineated by criteria where the FDR (adjusted p‐value) was below 0.05 and the absolute Log2 fold change between patient and control conditions exceeded 0.1. DEGs profiles from each cell type and subcluster were subjected to GO Enrichment Analysis. The resulting bobble plots illustrated the expression patterns of the enriched pathways across various cell clusters. The enrichment score served as a metric to quantify the overrepresentation of a gene set within a specific database of gene sets.

### Quantification and Statistical Analysis

Data are presented as mean ± SEM. Unpaired two‐tailed *t*‐tests, one‐way ANOVA, and two‐sided Fisher's exact tests were used to determine statistical significance. These analyses were conducted using GraphPad Prism software (version 10.0) and R software (version 4.3.1), respectively. Significance level as ^*^ *p* < 0.05, ^**^ *p* < 0.01, ^***^ *p* < 0.001. Statistical details of each experiment are shown in the figure legends.

### Data Availability Statement

The data supporting the results of this study are available within the paper. The raw bulk RNA‐seq and single‐cell RNA‐seq data are available from the Genome Sequence Archive (GSA) database under accession number HRA005966. Additionally, the DFO‐C2 and VFO‐C2 bulk RNA‐seq data are available under BioSample accessions HRS910741 and HRS910754, and the single‐cell RNA‐seq data are available under accessions HRS910767 and HRS910780 in HRA005215. Whole‐exome sequencing raw data of patients’ blood leukocytes and astrocytes derived from surgically resected brain tissue are available under accession number HRA008230.

## Conflict of Interest

The authors declare no conflict of interest.

## Author Contributions

J.X. and Y.K. contributed equally to this work. Z.S. and J.X. designed the experiments. J.X., Y.K., and Z.S. performed astrocyte reprogramming, organoid culture, and sequence of transcription profile of organoid. Y.K. performed organoid analysis. R.Z., J.X., and N.W. isolated patient astrocytes. J.X. and H.L. did single‐cell RNA sequence analysis. Z.S. and J.X. did RNA‐seq analysis. J.X. and Y.L. conducted the induction of cardioids. Z.S., J.X., Y.K., R.Z., and N.W. wrote the manuscript and data interpretations. Z.S., Z.L., Y.Y., X.Y., H.L., and J.D. reviewed data interpretations and manuscript contents. Z.S. supported this study financially.

## Supporting information



Supporting Information

Supplemental Table 1

Supplemental Video 1

Supplemental Video 2

Supplemental Video 3

Supplemental Video 4

Supplemental Video 5

Supplemental Video 6

Supplemental Video 7

## Data Availability

The data that support the findings of this study are available in the supplementary material of this article.
