# Supplementary material for: Personalized Human Astrocyte‐Derived Region‐Specific Forebrain Organoids Recapitulate Endogenous Pathological Features of Focal Cortical Dysplasia
Source: Adv Sci (Weinh). 2024 Dec 31;12(8):2409774. doi: 10.1002/advs.202409774 (PMC11848560; doi:10.1002/advs.202409774)
Supplement: Supplementary file 1 — Supporting Information [file ADVS-12-2409774-s008.docx]

**Extended Figures and Legends:**

**
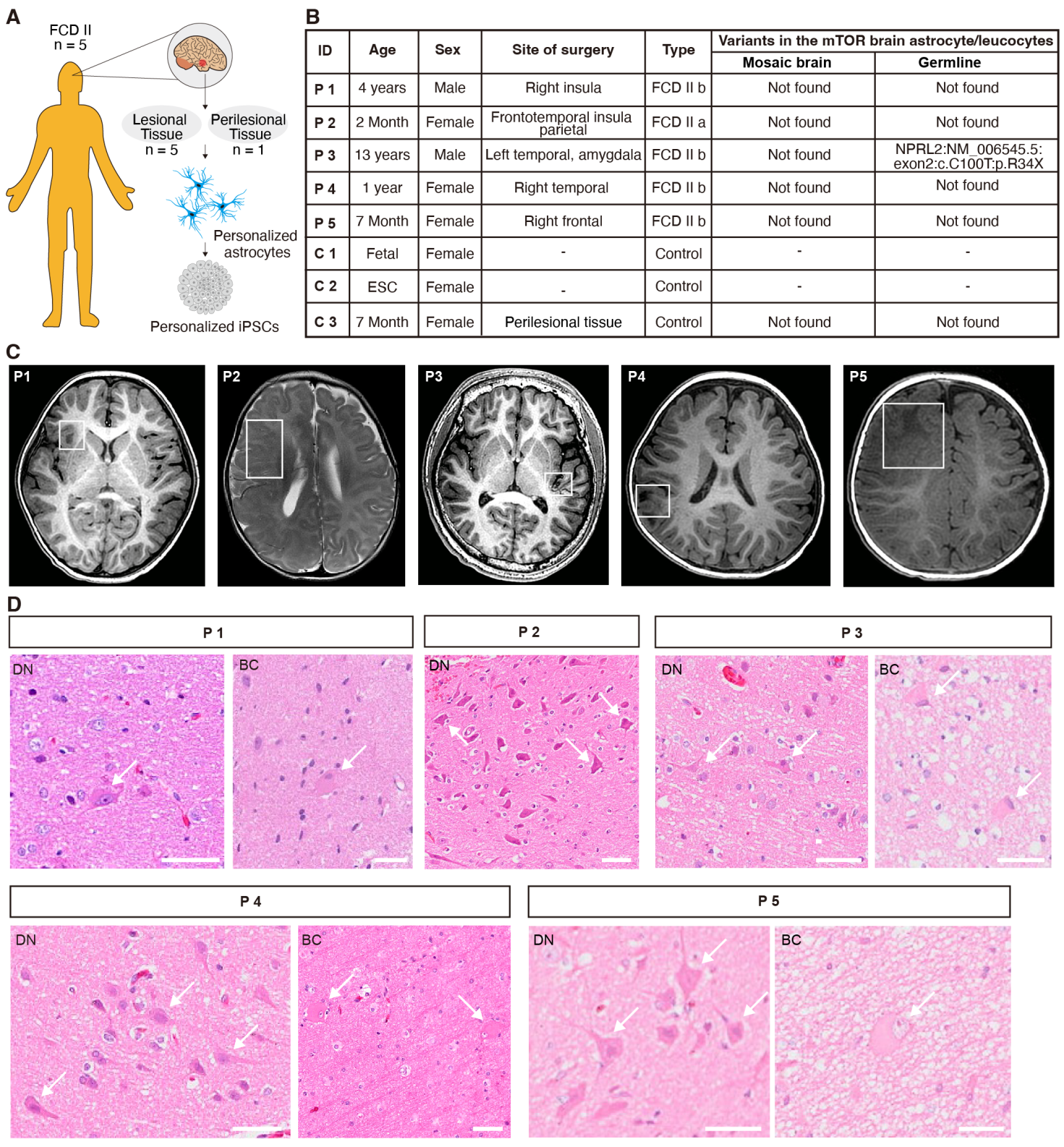
**

**Figures S1. The clinical information and pathological characterizations of FCD II patients.** A) Process diagram of collecting and establishing personalized iPSCs from FCD patients. B) Surgical information of FCD II patients and controls. C) Coronal T1-weighted MRI of five patients, the red square shows the epileptogenic zone. D) Hematoxylin-eosin staining results of brain tissues from five patients indicated abnormalities in the focal lesion. White arrows point to the abnormal dysmorphic neurons (DN) and balloon cells (BC). Scale bar, 50 μm.

**
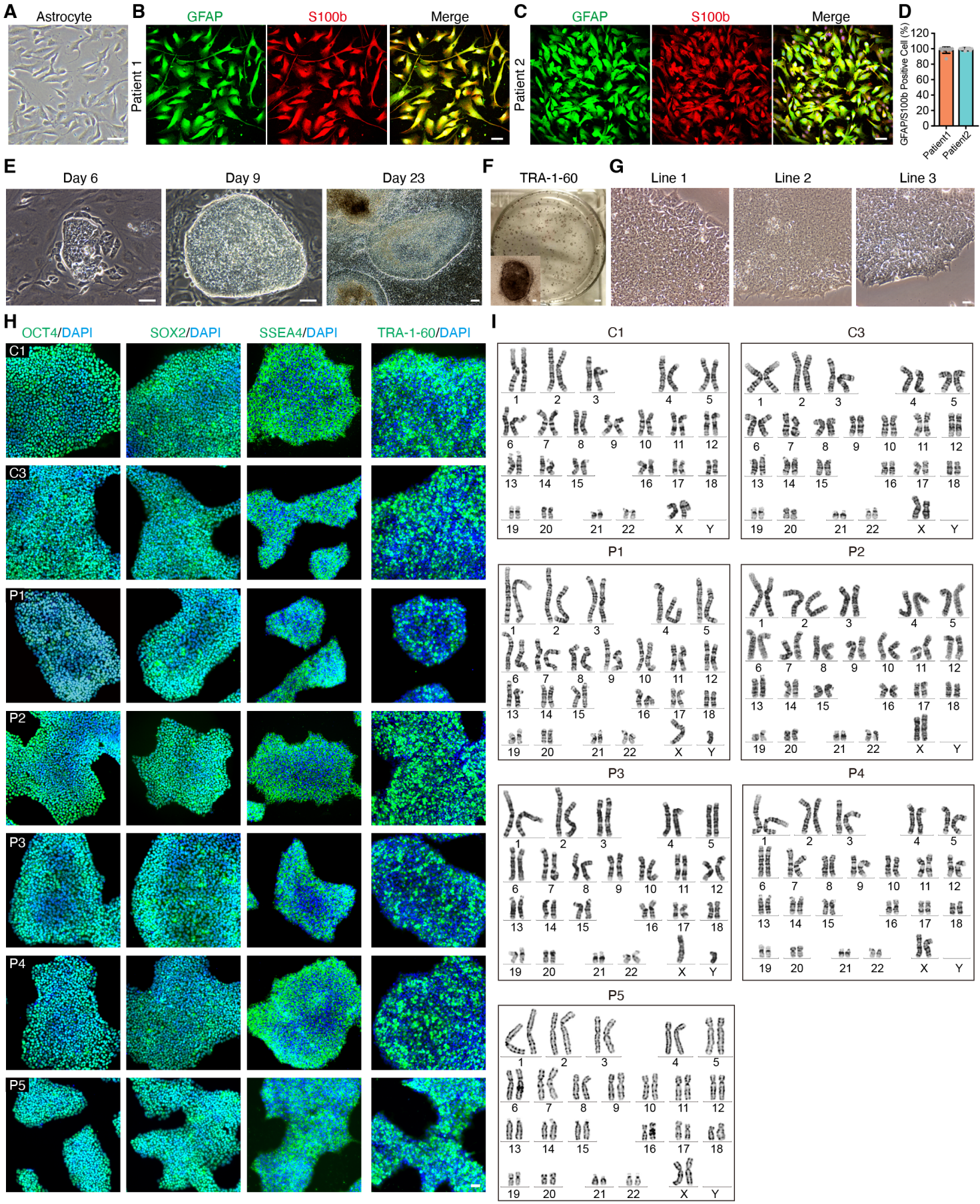
**

**Figures S2. Establishment of personalized astrocyte-derived iPSCs from patients.** A-C) Representative images of astrocytes purified from brain tissue excised from FCD patients (A) and characterizing astrocytes by immunostaining for GFAP and S100b (B and C). Scale bar, 50 μm. D) Quantitative analysis of the proportion of astrocytes derived from patients at several randomly chosen fields (n > 5 fields in each patient). Error bar is presented as mean ± SEM. E) Phase images showing clones at different time points (day 6, 9, 23) after electroporation reprogrammed factors into astrocytes from patients. Scale bar, left and center: 200 μm, right: 500 μm. F) DAB staining of the TRA-1-60 showed the pluripotency of reprogrammed clones at day 25. Scale bar, 2000 μm. The lower left corner is a zoomed-in image of a single clone. Scale bar, 100 μm. G) The selected different individualized iPS cell lines maintained a dense clonal morphology. Scale bar, 50 μm. H) Expression of pluripotent markers including OCT4, SOX2, SSEA4, and TRA-1-60 showing the pluripotency of personalized iPSCs. Scale bar, 50 μm. I) Karyotype analysis showing the genetics of the astrocyte-derived iPSCs.

**
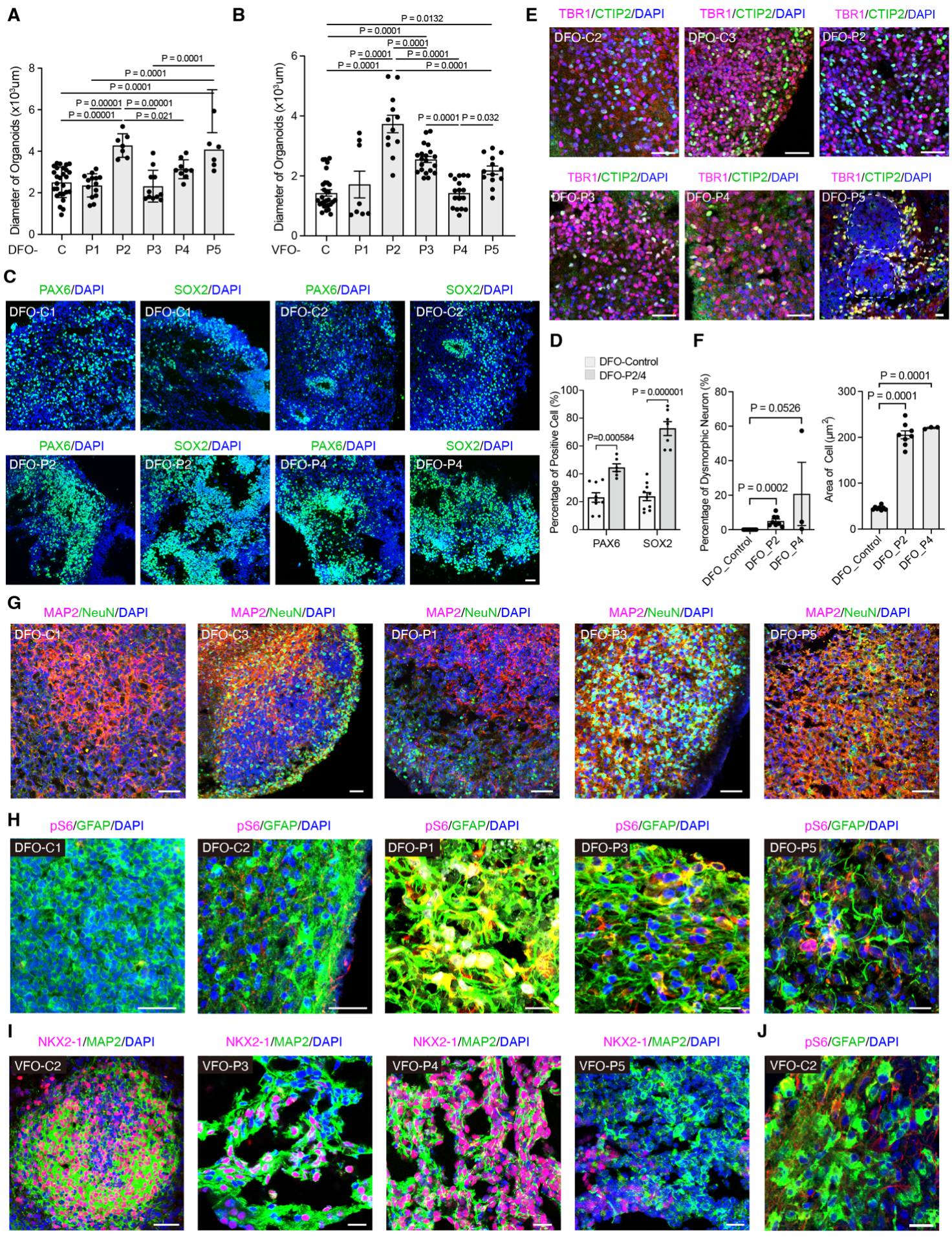
**

**Figures S3. Pathological characterization of dorsal and ventral forebrain organoids derived from FCD II patients.** A, B) Diameter size statistics of dorsal forebrain organoids (A) and ventral forebrain organoids (B) at 12 weeks (n = 3 independent experiments every patient and control). C) Immunostaining of DFOs expressing forebrain neural progenitors PAX6 and SOX2 at 6 weeks. Scale bar, 50 μm. D) Quantitative analysis of the percentage of PAX6 and SOX2 positive progenitors in DFO-P2 and DFO-P4 compared to controls (n = 3 slices staining). E) Immunostaining of DFOs expressing deep cortical glutamatergic neuron marker TBR1 and CTIP2 at 12 weeks. Scale bar, 50 μm. F) Quantitative analysis of the percentage of dysmorphic neurons and their size in DFO-P2 and DFO-P4 compared to controls (n = 3 slices staining). G) Neuronal marker MAP2 and NeuN staining for dysmorphic neurons in the DFO-C1 and DFO-C3, and the patient group, DFO-P1, DFO-P3, DFO-P5 at 12 weeks. Scale bar, 50 μm. H) Pathologic characterization of balloon cells by staining for the phospho-protein pS6 and the astrocyte marker GFAP in 12-week DFOs from control groups DFO-C1 and DFO-C2 and patient groups DFO-P1, DFO-P3, and DFO-P5. Scale bar, center: 20 μm; left and right: 50 μm. I) VFO-C2 and VFO-P3, VFO-P4, and VFO-P5 were stained at 6 weeks to identify expression of MGE neural progenitor cell (NPC) marker NKX2-1 and neuronal marker MAP2. Scale bar, 50 μm. J) pS6 and GFAP staining in 12-week VFO-C2. Scale bar, 50 μm. A, B, D, F) Error bars are presented as mean ± SEM; Ordinary one-way ANOVA were used to compare the significance of each group in A and B. The p-value was determined using an unpaired two-tailed t-test in D and F.

**
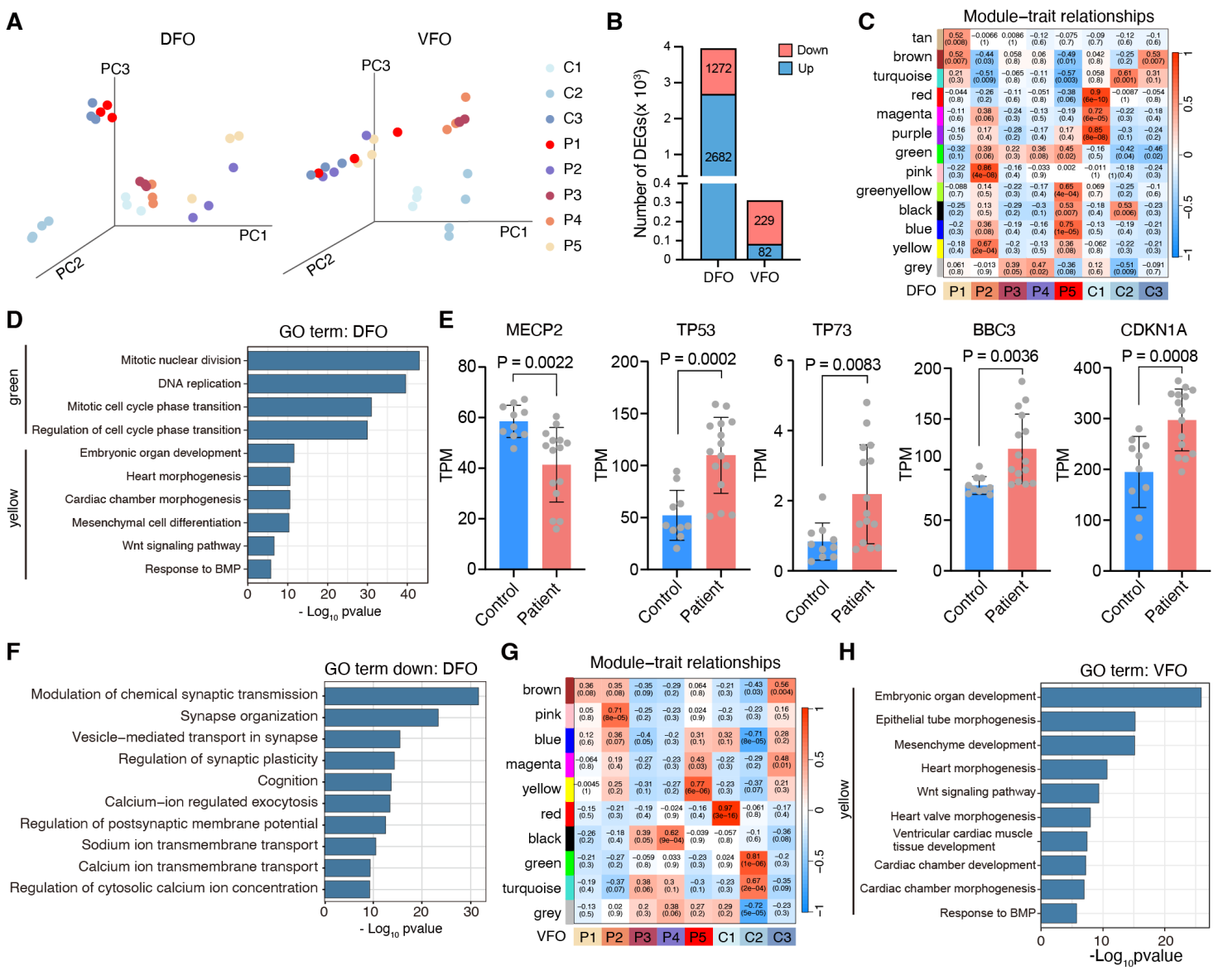
**

**Figures S4. Abnormal cardiomyocyte development and cellular senescence phenotypes appear in forebrain organoids derived from FCD patients.** A) 3D Scatterplot illustrates principal component analysis (PCA) of DFOs and VFOs from patients and controls (n = 3 bulk samples every patient and control). B) The differential gene expression analysis shows downregulated and upregulated genes in the patient group compared with controls (*P* < 0.05, |log_2_FC| > 0.58). C and D) Weighted correlation network analysis (WGCNA) of DFOs shows that the green and yellow module GO terms (D) primarily express genes in DFO-P2 and DFO-P5 (*P* < 0.05, |log_2_FC| > 0.58). E) Graphs illustrating the expression profiles of p53 pathway-associated genes in patient DFOs (n = 3 bulk samples every patient and control). Error bars are presented as mean ± SEM; The p-value was determined using an unpaired two-tailed t-test. F) GO term enrichment analysis of genes significantly downregulated (*P* < 0.05, |log_2_FC| > 0.58) in patient DFOs compared with controls. G and H) Weighted correlation network analysis (WGCNA) of VFOs shows that the yellow module GO term (H) primarily expresses genes involved in myocardial development in DFO-P2 and DFO-P5 (*P* < 0.05, |log_2_FC| > 0.58).


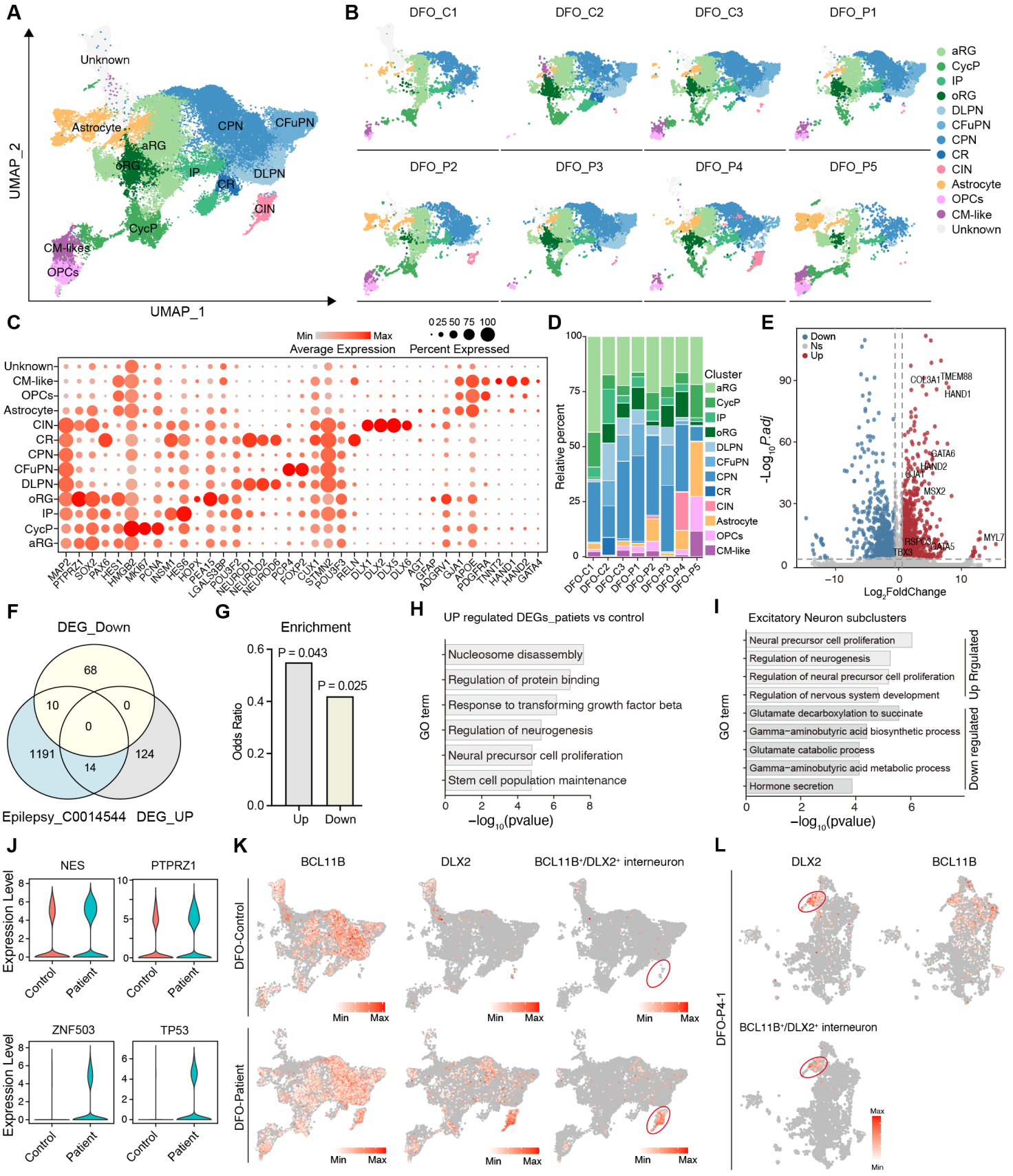


**Figures S5. scRNA-seq analysis of DFOs derived from FCD patients.** A) Visualized clustering of cells from all the DFOs after batch correction using UMAP. B) Cell clustering of DFOs from each control and patient was integrated using harmony and depicted using UMAP. C) Dot plots display the expression of selected marker genes across clusters, with the size of each circle indicating the percentage of cells expressing each gene within the cluster. D) The distribution of each subclusters in 12-weeks dorsal forebrain organoid. E) Volcano plots visualize the differential gene expression results in patient DFOs compared to controls. X and Y axes show average log_2_FC and -log_10_(P value). F) Venn diagram displays the overlap of differentially expressed genes (DEGs) in patient DFOs with the DisGeNET epilepsy Gene-Disease Association list (CUI: C0014544). G) Two-sided Fisher’s Exact Test was used to determine the enrichment for upregulated and downregulated genes of patient DFOs in epilepsy gene list. Odds ratio from the test are displayed along with Bonferroni-corrected *P* values. H) GO term enrichment analysis of genes significantly up and down regulated (P-adjust < 0.05, |pct.1-pct.2| > 0.1) in patient DFOs compared with controls. I) Perform a GO term enrichment analysis on genes that are significantly up-regulated or down-regulated (with P-adjust < 0.05 and |pct.1 - pct.2| > 0.1) in excitatory neurons including DLPN, cFuPN, CPN, and CR subclusters in patient-derived organoids (DFOs) compared to control samples. J) Violin plots display the relative expression levels of NES, ZNF503, PTPRZ1, and TP53 in DFOs from controls and patients. K, L) BCL11B and DLX2 expression, as well as their co-expression in cell clusters of DFOs from controls and patients, were visualized using UMAP (K). DFO-P4-1: a repeated single-cell sequencing of DFOs derived from patient 4 (L).

**
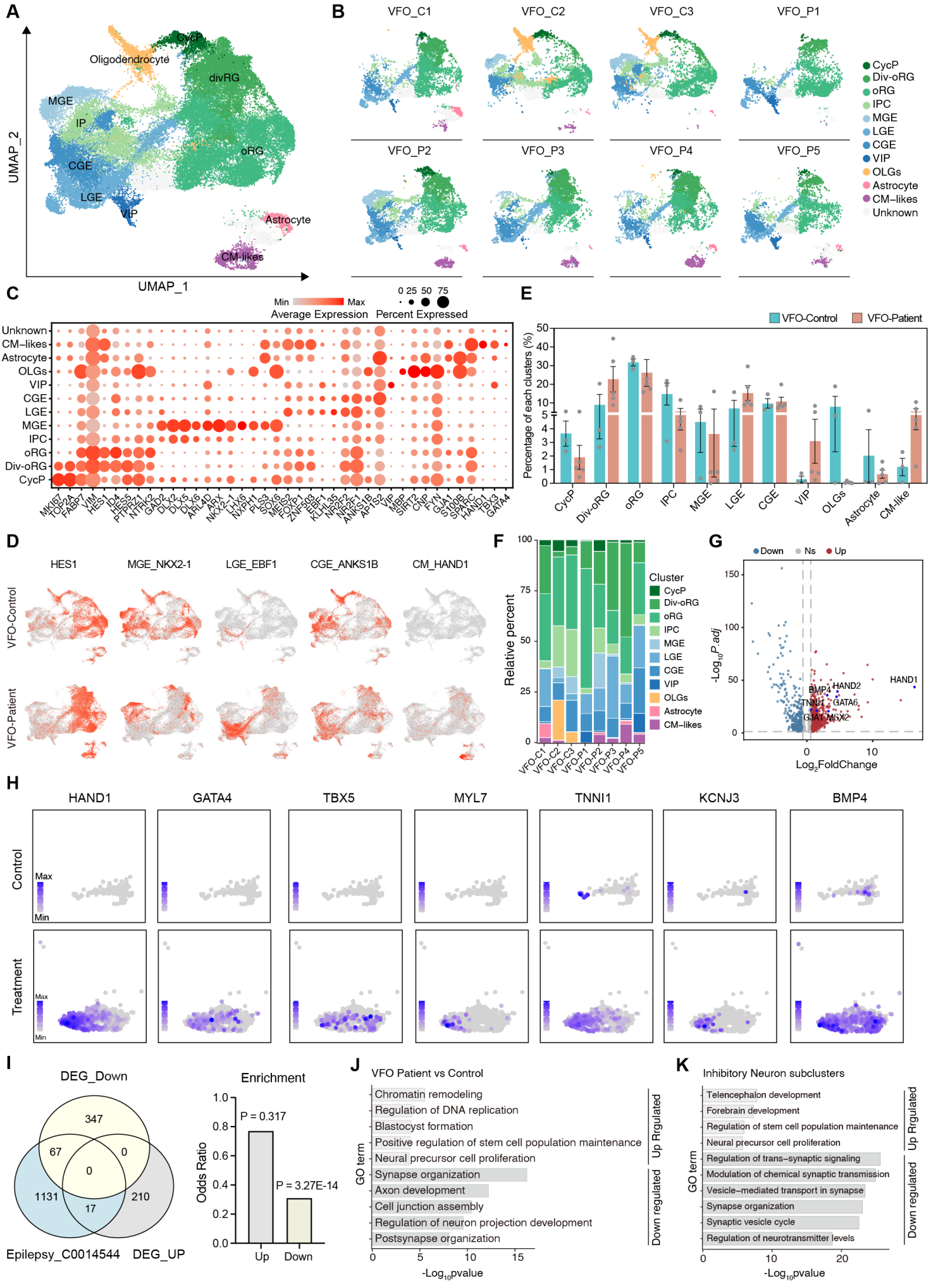
**

**Figures S6. scRNA-seq analysis of VFOs derived from FCD patients.** A) Visualized clustering of cells from all the VFOs after batch correction using UMAP. B) Cell clustering of VFOs from all controls and patients was integrated using Harmony and visualized separately with UMAP. C) Dot plots showing the expression of selected marker genes in each of the clusters. The size of the circle reveals the percentage of cells expressing each gene per cluster. D) Selected genes expressed in cell clusters of VFOs from controls and patients were visualized using UMAP. E) Separation of the data by patient and control status showed the proportion of each cluster in FCD II VFOs compared to controls. F) The distribution of each subclusters in 12-weeks ventral forebrain organoid. G) Volcano plots visualize the differential gene expression results in patient VFOs compared to controls, with some genes associated with cardiac mesoderm development labeled. H) Subclusters of CM-like cells derived from patient VFOs exhibit dysregulated expression of genes crucial for cardiomyocyte development, including the cardiac transcription factors HAND1, GATA4, and TBX5, alongside myocardial structural proteins and ion channel protein such as MYL7, TNNI1, and KCNJ3, as well as the cell development signaling molecule BMP4. I) Venn diagram displays the overlap of differentially expressed genes (DEGs) in patient VFOs with the DisGeNET epilepsy Gene-Disease Association list (CUI: C0014544). And Two-sided Fisher’s Exact Test was used to determine the enrichment for upregulated and downregulated genes of patient VFOs in epilepsy gene list. Odds ratio from the test are displayed along with Bonferroni-corrected *P* values. J) GO term enrichment analysis of genes significantly up-regulated and down-regulated (P-adjust < 0.05, |pct.1-pct.2| > 0.1) in patient VFOs compared with controls. K) GO term enrichment analysis on genes that are significantly up-regulated or down-regulated (with P-adjust < 0.05 and |pct.1 - pct.2| > 0.1) in inhibitory neurons including MGE, LGE, CGE, and VIP subclusters in patien derived organoids (DFOs) compared to control samples.

**
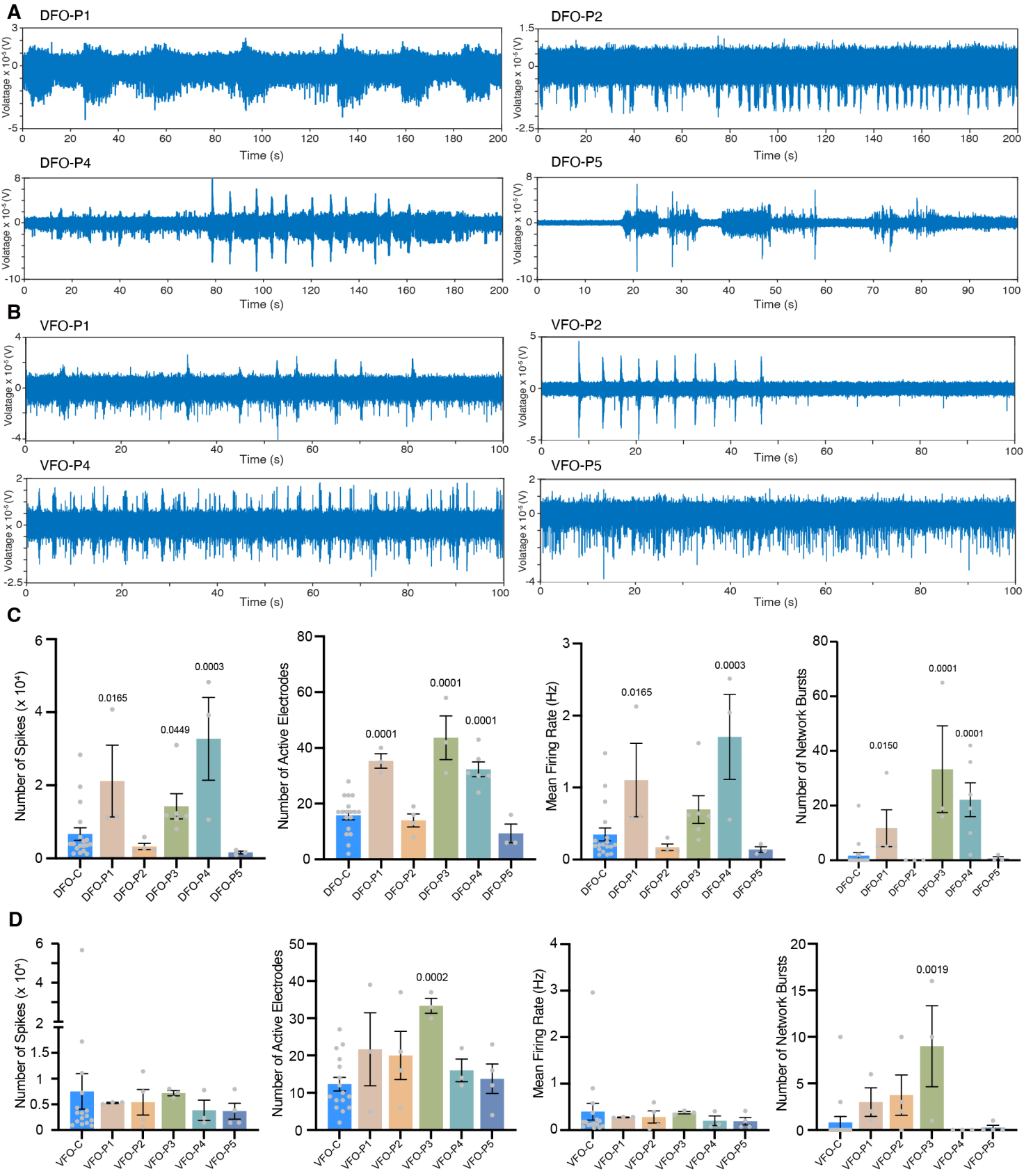
**

**Figures S7. Epileptiform burst firing was observed in both FCD patient-derived DFOs and VFOs.** A, B) Electrographic seizure-like activities of P1, P2, P4, and P5-derived DFOs (A) and VFOs (B) using MEA recording. C, D) Graphs showing number of spikes and active electrodes, mean firing rate, number of network bursts in FCD II DFOs (C) and VFOs (D) compared with controls (n=3 independent experiments every patient and control). Error bars are presented as mean ± SEM; The p-value was determined using an unpaired two-tailed t-test.

**
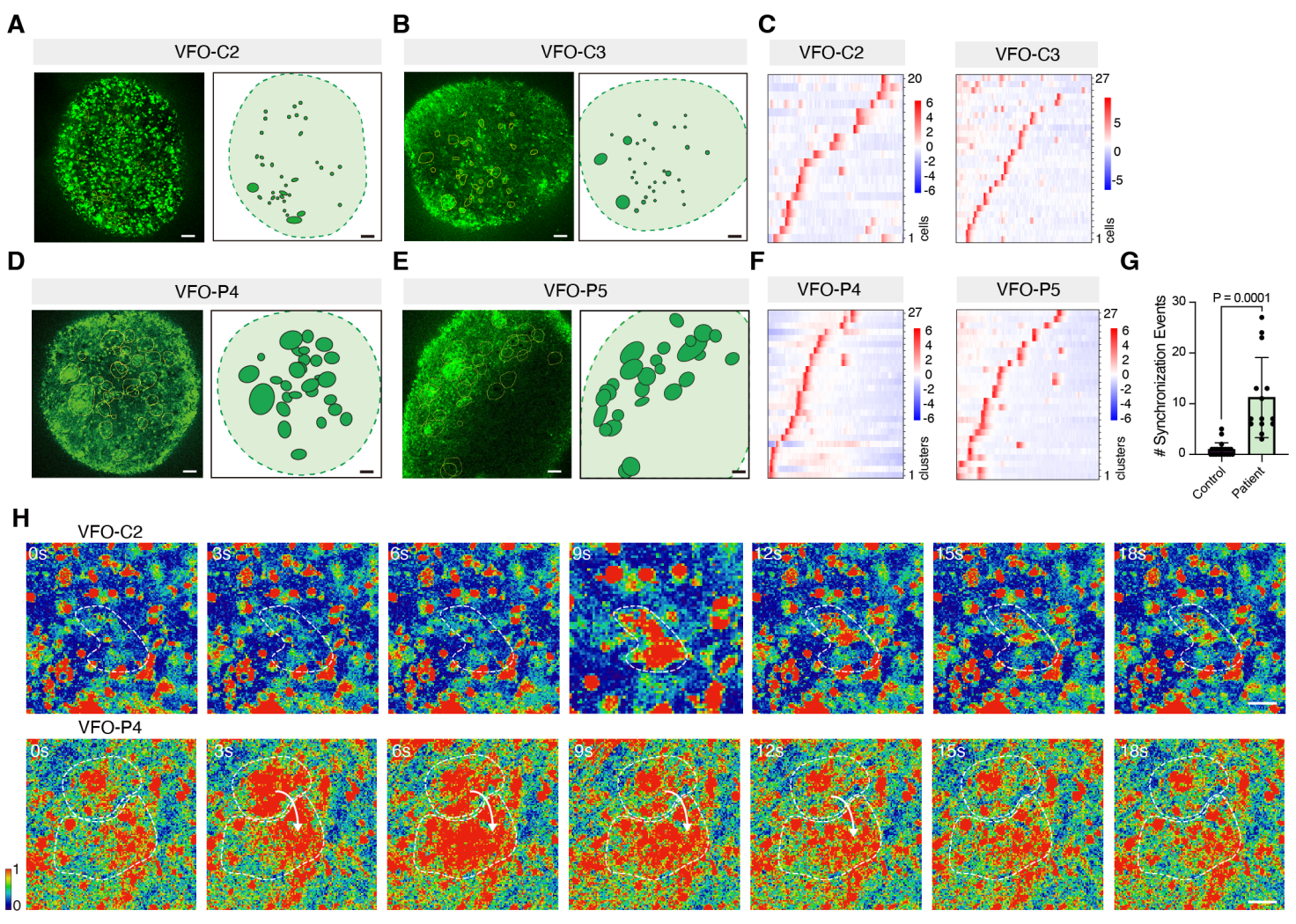
**

**Figures S8. Assembling neuronal hyperactivity in VFOs derived from FCD patients.** A-C) Calcium image showing firing activity of control VFO-C2 and VFO-C3, along with a heat map illustrating single-cell calcium transients recorded for 100 cycles (C). Scale bars, 100 μm. D-F) Calcium image showing firing activity of patient VFO-P4 and VFO-P5 and heatmap showing cell cluster calcium transient of 100 cycles recorded (F). Scale bars, 100 μm. G) Higher frequency of synchronization events in patient-derived VFOs compared with controls (n=3 independent experiments every patient and control). Error bar is presented as mean ± SEM; The p-value was determined using an unpaired two-tailed t-test. H) Time-series plots showing spontaneous calcium transients in single cells of VFO-C2 and the pattern of spontaneous synchronized clustered calcium activity in VFO-P5 at 3-second intervals, white arrows showing the direction of fluctuations in synchronized clustered calcium activity transmission. Scale bar, 100 μm.

**
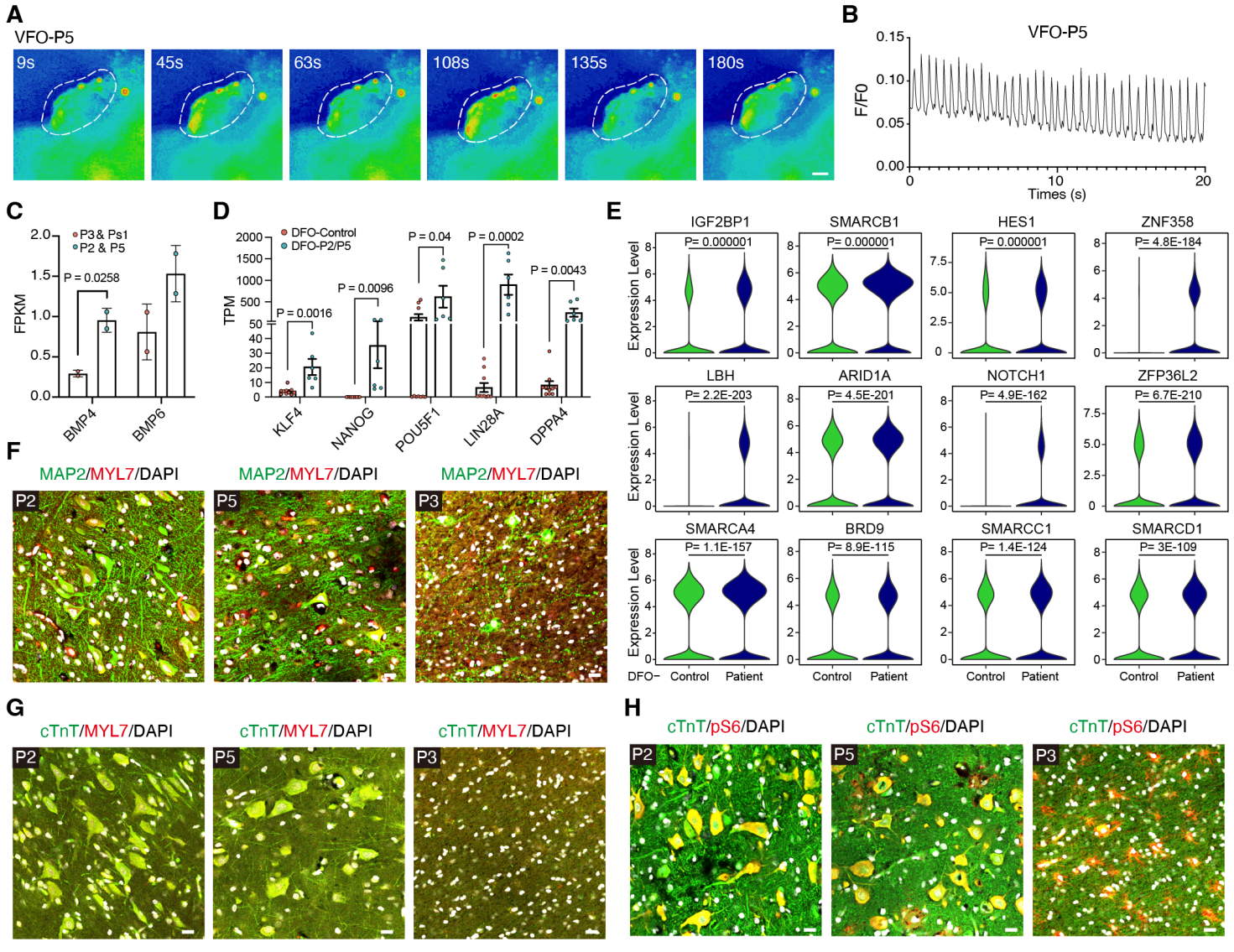
**

**Figures S9. Pluripotent gene expression in patient-DFOs and abnormal cardiomyocyte-specific gene profiles in pathological brain tissue of FCD.** A) Fluo-4-AM fluorescence staining of VFO-P5 neuro-cardiac organoids for calcium imaging. Representative graphs represent the fluorescence intensity of myocardial beating-induced calcium transients over time. Scale bar, 50 μm. B) Calcium transients were counted in VFO-P5 over a 20-second period. C) Transcriptome sequencing of focal brain tissue from patients P2, P5 versus P3 and P1 parafocal brain tissue (Ps1) showing differential expression of BMP4, BMP6 (n = 1 surgical specimen each patient). D) Sequencing analysis of the 6-week DFOs transcriptome revealed that the pluripotency genes KLF4, NANOG, POU5F1, LIN28A, and DPPA4 were significantly upregulated in DFO-P2 and DFO-P5 compared to controls (n = 3 independent experiments every patient and control). E) Violin plots display the relative expression levels of stem cell maintenance genes in DFOs from controls and patients at 12 weeks, based on single-cell sequencing. F-H) Representative staining shows myocardial structural protein MYL7 and neuronal marker MAP2 in focal brain tissue from patients P2, P5, and P3 (F). Additionally, myocardial structural protein cTnT co-expresses with MYL7 (G), and cTnT co-expresses with phosphorylated S6 in the focal brain tissue from patients P2, P5, and P3. Scale bar, 20 μm. C, D, E) Error bars are presented as the mean ± SEM in C and D. The p-value was determined using an unpaired two-tailed t-test for figures C, D, and E.


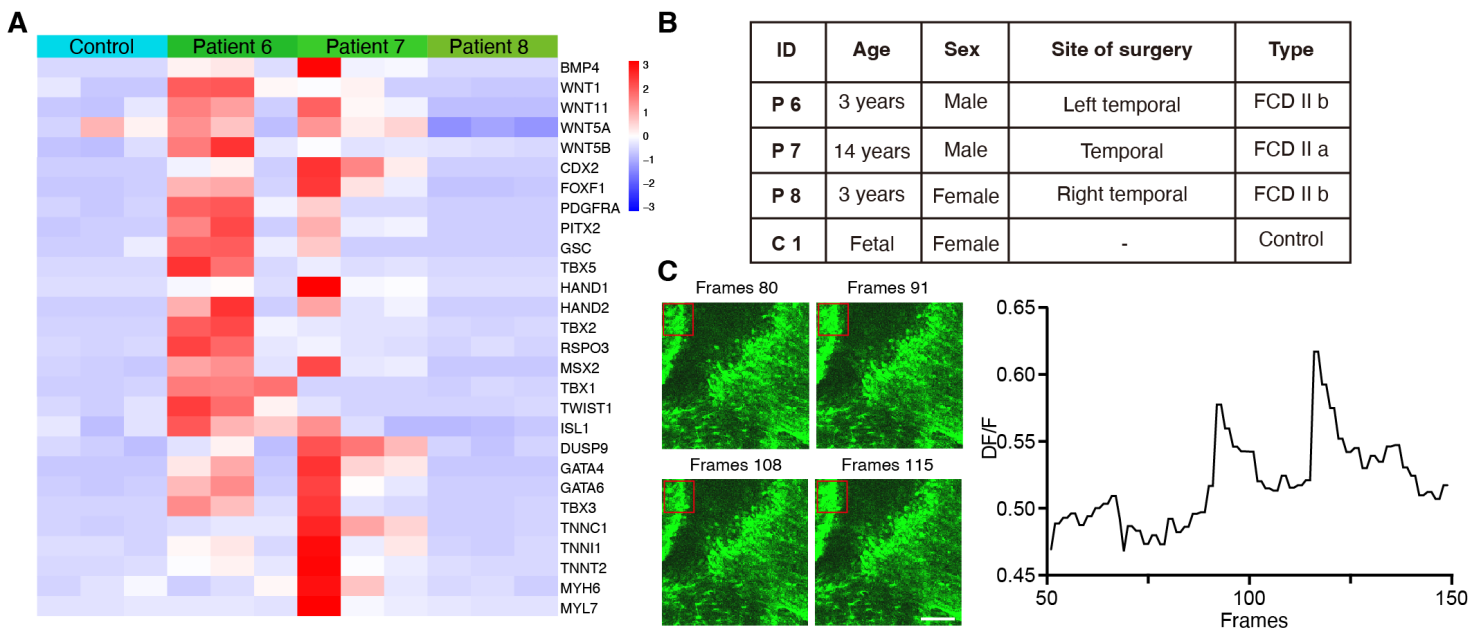


**Figures S10. Confirmation of cardiomyocyte heterotopia in three additional FCD II patient-derived forebrain organoids.** A) Comparison of differentially expressed genes between FCD II forebrain organoids and controls revealed a significant upregulation of cardiomyocyte development marker genes in forebrain organoids derived from patients 6 and 7. B) Surgical information of patients and control in (A). C) Fluo-4-AM fluorescence staining was performed on DFO-P6 neuro-cardiac organoids for calcium imaging. The statistics indicate fluorescence intensity changes consistent with rhythmic calcium activity similar to that observed in cardiomyocytes. Scale bar, 100 μm.


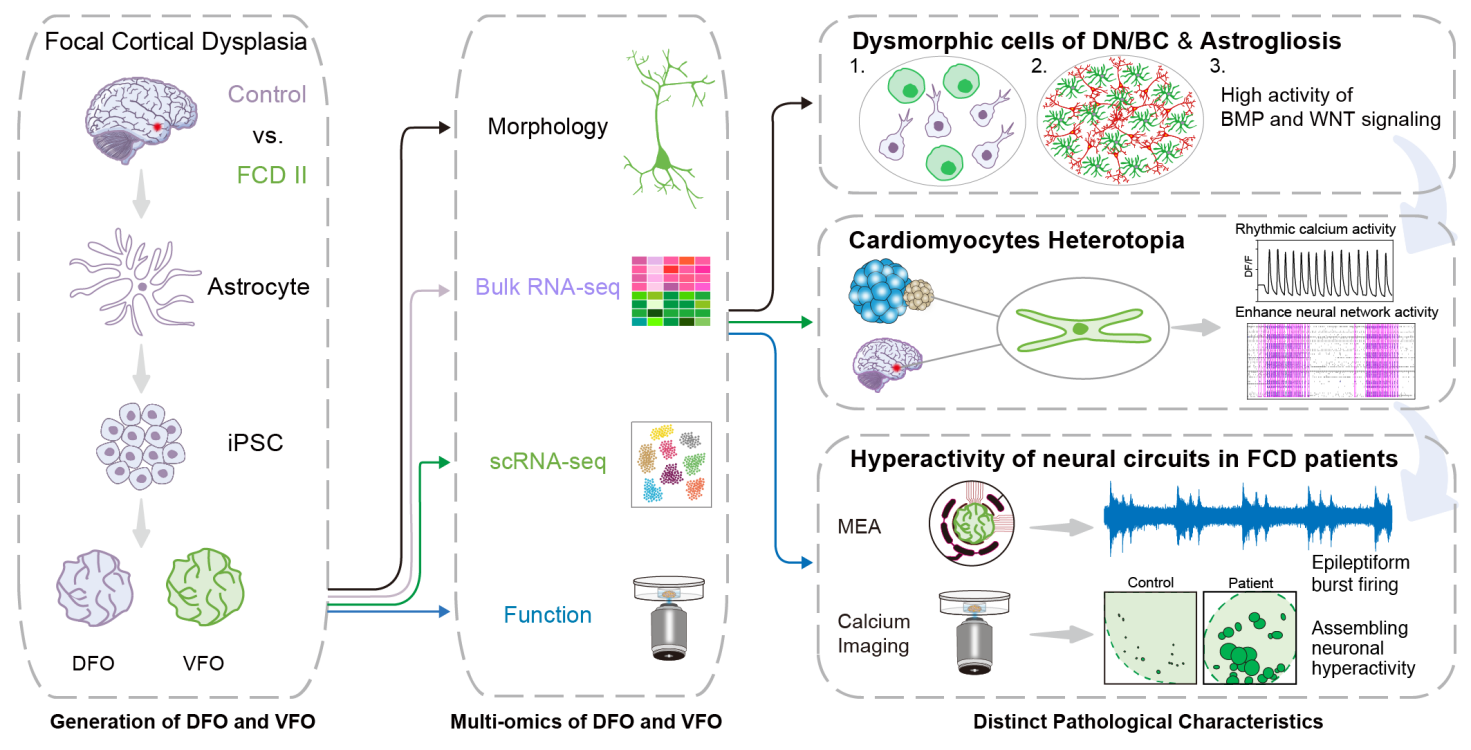


**Figures S11. Multiple pathological features in personalized human astrocyte-derived region-specific forebrain organoids.** Conceptual schematic diagram underlining the main results in this study.
